# Durvalumab with platinum-pemetrexed for unresectable pleural mesothelioma: survival, genomic and immunologic analyses from the phase 2 PrE0505 trial

**DOI:** 10.1038/s41591-021-01541-0

**Published:** 2021-11-08

**Authors:** Patrick M. Forde, Valsamo Anagnostou, Zhuoxin Sun, Suzanne E. Dahlberg, Hedy L. Kindler, Noushin Niknafs, Thomas Purcell, Rafael Santana-Davila, Arkadiusz Z. Dudek, Hossein Borghaei, Mara Lanis, Zineb Belcaid, Kellie N. Smith, Archana Balan, James R. White, Christopher Cherry, I. K. Ashok Sivakumar, Xiaoshan M. Shao, Hok Yee Chan, Dipika Singh, Sampriti Thapa, Peter B. Illei, Drew M. Pardoll, Rachel Karchin, Victor E. Velculescu, Julie R. Brahmer, Suresh S. Ramalingam

**Affiliations:** 1 The Sidney Kimmel Comprehensive Cancer Center, Johns Hopkins University School of Medicine, Baltimore, MD USA; 2 The Bloomberg-Kimmel Institute for Cancer Immunotherapy, Johns Hopkins University School of Medicine, Baltimore, MD USA; 3 ECOG-ACRIN Biostatistics Center, Boston, MA USA; 4 Frontier Science Foundation, Boston, MA USA; 5 Boston Children’s Hospital, Boston, MA USA; 6 University of Chicago Medicine, Chicago, IL USA; 7 University of Washington School of Medicine & Fred Hutchinson Cancer Research Center, Seattle, WA USA; 8 Metro-Minnesota Community Oncology Research Consortium, St. Louis Park, MN USA; 9 Fox Chase Cancer Center, Philadelphia, PA USA; 10 Institute for Computational Medicine, Johns Hopkins University, Baltimore, MD USA; 11 Department of Biomedical Engineering, Johns Hopkins University, Baltimore, MD USA; 12 Department of Pathology, Johns Hopkins University School of Medicine, Baltimore, MD USA; 13 Department of Hematology and Medical Oncology, Emory University School of Medicine, Winship Cancer Institute, Atlanta, GA USA

**Keywords:** Mesothelioma, Cancer genomics, Cancer immunotherapy, Tumour immunology, Phase II trials

## Abstract

Mesothelioma is a rare and fatal cancer with limited therapeutic options until the recent approval of combination immune checkpoint blockade. Here we report the results of the phase 2 PrE0505 trial (NCT02899195) of the anti-PD-L1 antibody durvalumab plus platinum-pemetrexed chemotherapy for 55 patients with previously untreated, unresectable pleural mesothelioma. The primary endpoint was overall survival compared to historical control with cisplatin and pemetrexed chemotherapy; secondary and exploratory endpoints included safety, progression-free survival and biomarkers of response. The combination of durvalumab with chemotherapy met the pre-specified primary endpoint, reaching a median survival of 20.4 months versus 12.1 months with historical control. Treatment-emergent adverse events were consistent with known side effects of chemotherapy, and all adverse events due to immunotherapy were grade 2 or lower. Integrated genomic and immune cell repertoire analyses revealed that a higher immunogenic mutation burden coupled with a more diverse T cell repertoire was linked to favorable clinical outcome. Structural genome-wide analyses showed a higher degree of genomic instability in responding tumors of epithelioid histology. Patients with germline alterations in cancer predisposing genes, especially those involved in DNA repair, were more likely to achieve long-term survival. Our findings indicate that concurrent durvalumab with platinum-based chemotherapy has promising clinical activity and that responses are driven by the complex genomic background of malignant pleural mesothelioma.

## Abstract (web summary)

In a phase 2 trial, the combination of chemotherapy with durvalumab, an anti-PD-L1 antibody, exhibited promising clinical activity in patients with previously untreated, unresectable mesothelioma, with additional analyses providing insights into genomic and immunologic features potentially associated with response.

## Main

Malignant pleural mesothelioma (MPM) affects more than 30,000 people worldwide each year and is fatal in nearly all cases^1^. Exposure to asbestos and consequent chronic inflammation in the pleural cavity is responsible for most cases, with a typical disease latency of 30–40 years, especially in the context of co-occurring defects in DNA damage repair and germline cancer predisposition syndromes^2–4^. For over 15 years, cisplatin and pemetrexed combination chemotherapy was the only approved systemic therapy; this approval was based on a phase 3 study that showed an improvement in survival from 9.3 months with cisplatin alone to 12.1 months with the combination^5^. With the exception of bevacizumab (which has not received regulatory approval in the United States), adding novel agents to platinum doublet chemotherapy has not improved survival^6–9^. Recently, several phase 2 studies reported on the efficacy of single-agent PD-1 inhibitors in chemotherapy-pretreated MPM^10–13^. Following the non-small-cell lung cancer paradigm, where the combination of first-line chemotherapy with PD-1 pathway blockade has become a standard approach for advanced disease^14^, chemo-immunotherapy is currently being explored in MPM. In the first-line setting, the phase 2 DREAM study of durvalumab with chemotherapy achieved its primary endpoint of progression-free survival (PFS) at 6 months and showed the regimen to be tolerable and active in this setting^15^. Furthermore, the combination of the anti-CTLA-4 antibody ipilimumab with the anti-PD-1 antibody nivolumab was shown to improve survival for previously untreated patients when compared to chemotherapy, with robust efficacy being limited to non-epithelioid histology^16^.

Although several studies have expanded understanding of the genomic landscape of MPM and identified putative actionable alterations, these have not been translated to therapeutic progress^17–19^. More than 50% of MPMs carry germline or somatic mutations in genes involved in DNA repair and homologous recombination^3,20^. BAP1, a nuclear ubiquitin carboxyterminal hydrolase, has been reported to be frequently mutated in the germline and tumor cells of patients with MPM^17,18,20^. Heterozygous germline *BAP1* alterations predispose to mesothelioma, especially in the context of asbestos exposure^21^, and, similarly, germline *BLM* mutations might increase susceptibility to asbestos carcinogenesis and emergence of mesothelioma^22^. Inactivation of tumor suppressor genes, such as *BAP1*, *NF2*, *CDKN2A*, *TP53* and *SETD2*, by sequence or structural alterations, is thought to be the predominant oncogenic mechanism for MPM^17^. Notably, MPM harbors a low tumor mutation burden (TMB) of fewer than two non-synonymous mutations per megabase^17,18^ and has, therefore, been considered a tumor with low neoantigen-driven immunogenicity. Nevertheless, the promising clinical efficacy of immune checkpoint blockade for MPM calls for in-depth genomic and functional analyses to investigate the mechanisms of therapeutic response and resistance. In this study, we investigated the combination of durvalumab with platinum-based chemotherapy in a phase 2 clinical trial, to establish safety and efficacy and explore genomic and immunologic features of response in patients with unresectable MPM.

## Results

### Study design

**Extended Data Fig. 1:**
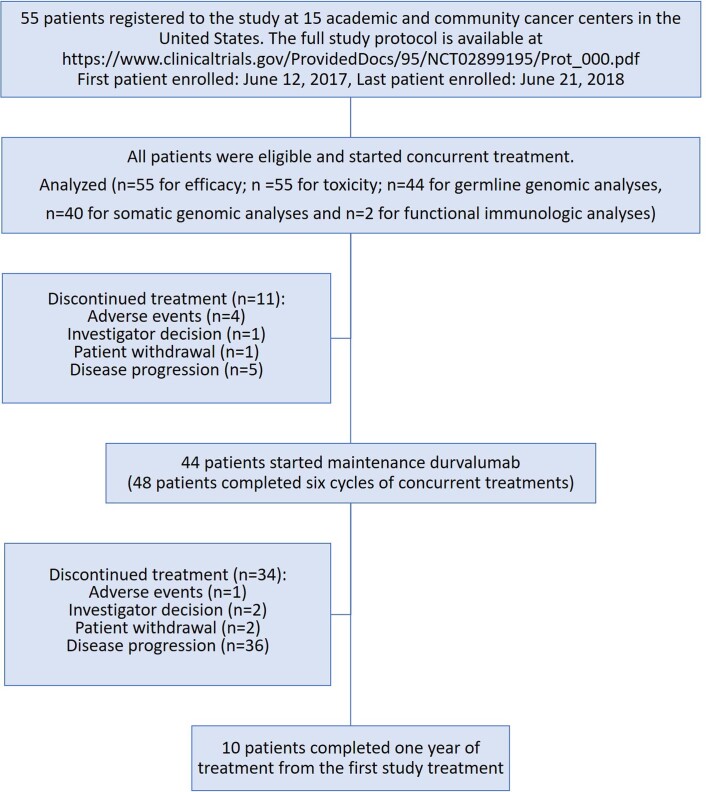
CONSORT trial diagram for the PrE0505 study. Outline of number of patients available for analyses.

PrE0505, a phase 2, single-arm, multicenter study, enrolled patients with previously untreated, unresectable MPM (NCT02899195; Extended Data Fig. 1). Patients received durvalumab (at a fixed dose of 1,120 mg intraveneously) given once every 3 weeks in combination with pemetrexed and cisplatin at their standard doses for up to six cycles. Substitution of carboplatin for cisplatin was permitted on cycle 1 for patients with a glomerular filtration rate (GFR) of ≥45 ml min^−1^ but ≤60 ml min^−1^, for patients with another documented contraindication to cisplatin (for example, hearing loss) or because of cisplatin toxicity during treatment. Patients with stable or responding tumors after concurrent therapy continued on maintenance durvalumab for a maximum duration of 1 year from the first study treatment. A protocol-defined safety review was performed after enrollment of the first six and 15 patients. Pre-specified dose-limiting toxicities during the safety run-in period included any immune-mediated adverse event of grade 4 or higher or any non-resolving, immune-mediated adverse event of grade 3 or higher during the first two cycles of therapy.

**Extended Data Fig. 2:**
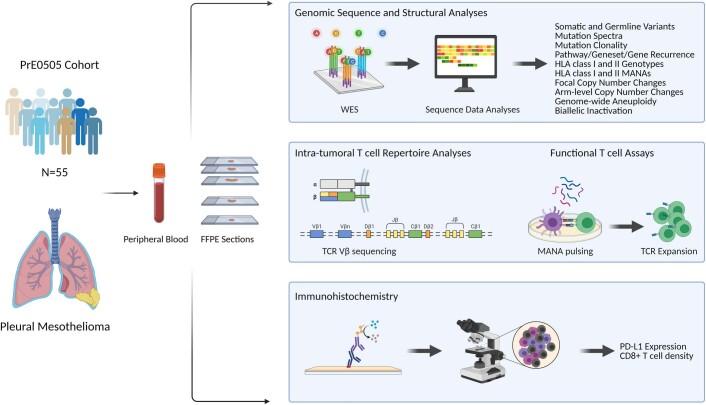
Analyses Overview. Whole exome sequencing data were analyzed to determine somatic sequence and focal copy number changes as well as germline variants in cancer predisposing genes and HLA class I and II haplotypes. Mutational spectra and enrichment in individual genes and gene sets were assessed. Genome-wide copy number analyses were performed to estimate genome aneuploidy, number of copy number breakpoints, homologous recombination deficiency as well as utilized to compute mutation cellular fractions. Computational predictions of mutation-associated neoantigens and immunogenic mutations were coupled with analyses of the intratumoral T cell repertoire and functional analyses of neopeptides-stimulated autologous peripheral T cells. PD-L1 and CD8+T cell expression was assessed by immunohistochemistry. This figure was developed using BioRender under a full license.

The primary endpoint of the study was overall survival (OS), defined as time from study registration to death from any cause. Patients last known to be alive were censored at their date of last follow-up. We assumed a null hypothesis that the median OS with chemo-immunotherapy would be equal to the OS of 12 months with pemetrexed/cisplatin alone (historical control). The total planned enrollment of 55 patients (50 eligible) with 32 events allowed for 90% power to detect a 37% reduction in the OS hazard rate of 0.058–0.037 based on Wald test for the log failure rate parameter using a one-sided type I error rate of 10%. This would correspond to a 58% improvement in the median OS from 12 to 19 months^4^. Secondary endpoints included PFS, best objective response and toxicity (Methods). PFS was defined as the time from study registration to documented disease progression or death from any cause, whichever occurred first. Patients who did not experience an event of interest were censored at the date they were last known to be alive and progression-free. Exploratory endpoints included investigating the genomic and immunologic underpinnings of response to chemo-immunotherapy; to this end, we performed whole exome sequencing (WES), coupled with genome-wide focal and large-scale copy number aberration analysis and sequence deconvolution (Methods). In parallel, we evaluated the intra-tumoral T cell repertoire and functional neoantigen-specific T cell responses (Methods and Extended Data Figs. 1 and 2).

### Participants

Between 12 June 2017 and 21 June 2018, PrE0505 enrolled 55 patients at 15 academic and community cancer centers in the United States. Eligible patients were 18 years of age or older and had histologically unselected MPM that was deemed to be surgically unresectable, an Eastern Cooperative Oncology Group (ECOG) performance status score of 0 or 1, adequate organ function including GFR of ≥45 ml min^−1^ and measurable disease by Response Evaluation Criteria in Solid Tumors (RECIST) 1.1 modified for pleural mesothelioma^23,24^. Key exclusion criteria were immunodeficiency, ongoing systemic immunosuppressive therapy, active autoimmune or infectious disease and clinically significant concurrent cancer. Demographics and disease characteristics are summarized in Table 1; median age was 68 years (range, 35–83 years), most patients were male (82%) and 75% of tumors were of epithelioid histology. Patients who had continued clinical benefit by investigator assessment (*n* = 20) were allowed to continue on treatment past radiographic progression.

**Table 1:** Demographic characteristics of study participants

Characteristic	Number (total *n* = 55)	Range or (%)
Age (years)	68 (median)	35–83
Sex		
Female	10	(18.2)
Male	45	(81.8)
Ethnicity		
Caucasian	47	(85.5)
African American	2	(3.6)
Other	6	(10.9)
ECOG performance status		
0	23	(41.8)
1	32	(58.2)
Histologic subtype		
Epithelioid	41	(74.5)
Sarcomatoid	7	(12.7)
Biphasic	6	(10.9)
Desmoplastic	1	(1.8)

### Efficacy analyses

**Fig. 1:**
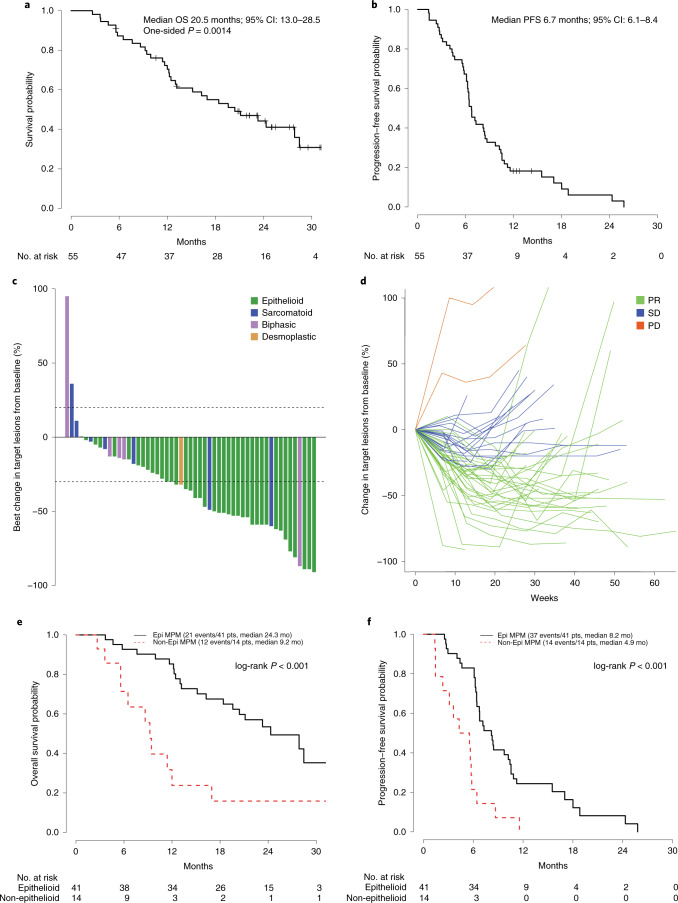
Outcomes with chemo-immunotherapy in unresectable MPM. **a**, Kaplan–Meier curve of OS in patients treated with durvalumab and platinum plus pemetrexed (*n* = 55). One-sided *P* value based on the Wald test for the log failure rate parameter is *P* = 0.0014, indicating significantly longer OS than the historical control of 12 months. **b**, Kaplan–Meier curve of PFS in patients treated with durvalumab and platinum plus pemetrexed (*n* = 55). **c**, Waterfall plot of best change in target lesions to treatment by histological subtype, based on maximal percentage of tumor reduction from baseline (*n* = 53 patients). Two patients without follow-up measurements in targeted lesions (one with best response unevaluable, the other with best response PD) were excluded. **d**, Spider plot of change in target lesions over time (*n* = 53 patients); notably, four patients had continued response or SD at the time of analysis. Two patients without follow-up measurements in targeted lesions (one with best response unevaluable, the other with best response PD) were excluded. **e**, Kaplan–Meier curves of OS according to histology; two-sided *P* value with significance level set at 0.05. **f**, Kaplan–Meier curves of PFS according to histology; two-sided *P* value with significance level set at 0.05. Epi, epithelioid. Source data

All patients were included in the eligible population for efficacy analyses. The median follow-up was 24.2 months at the time of this analysis, with 33 death events. The median OS for all patients enrolled was 20.4 months (95% confidence interval (CI): 13.0–28.5, 80% CI: 15.1–27.9) and was significantly longer than the historical control of 12 months (one-sided *P* = 0.0014; Fig. 1a), with an observed hazard rate of 0.034. The estimated percentages of patients alive at 6, 12 and 24 months were 87.2%, 70.4% and 44.2%, respectively. Median PFS was 6.7 months (95% CI: 6.1–8.4, 80% CI: 6.3–8.2; Fig. 1b). The estimated percentages of patients alive and progression-free at 6, 12 and 24 months were 67.3%, 18.2% and 6.1%, respectively. The objective response rate (ORR) was 56.4% (95% CI: 42.3–69.7% and 80% CI: 46.8–65.5%). No patients had a complete response (CR); 31 patients showed partial response (PR); and 20 patients had stable disease (SD). One patient was unevaluable for response owing to missing follow-up disease assessments, and three patients had progressive disease (PD) as best response (Fig. 1c,d). In a non-predefined subgroup analysis, a significant difference in ORR, PFS and OS was noted by histology. Patients with epithelioid tumors had a higher ORR than patients with non-epithelioid tumors (65.9% versus 28.6%, *P* = 0.03). Similarly, patients with epithelioid MPM had significantly longer median OS (24.3 months versus 9.2 months, hazard ratio (HR) = 0.27, 95% CI: 0.13–0.57, *P* < 0.001; Fig. 1e) and PFS (8.2 months versus 4.9 months, HR = 0.30, 95% CI: 0.16–0.58, *P* < 0.001; Fig. 1f) compared to patients in the non-epithelioid MPM group.

### Safety

**Extended Data Fig. 3:**
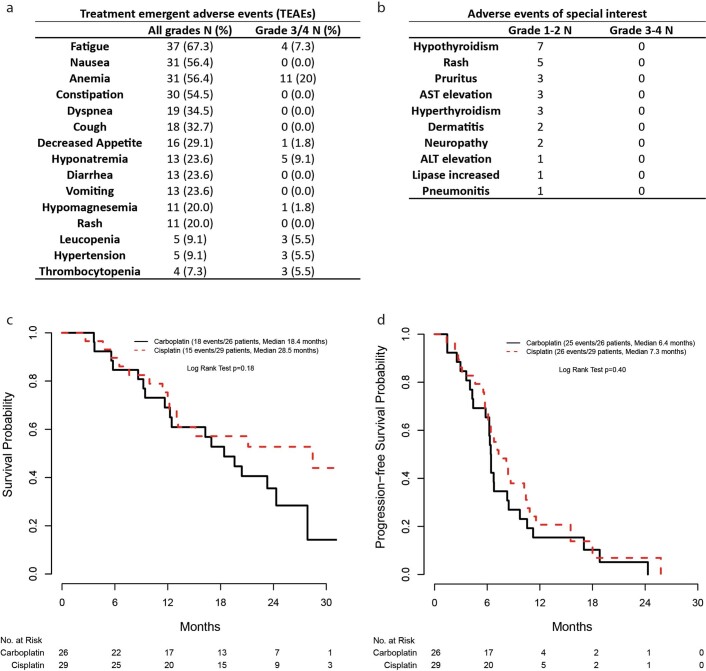
Treatment emergent adverse events and outcome by platinum agent used. (**a**) All grades and grade 3/4 treatment emergent adverse events; events reported in greater than 20% subjects or grade 3/4 events reported in greater than 5% of patients, worst grade reported per patient (CTCAE Version 4.03) (**b**) Adverse events of special interest, investigator reported assessment and attribution as possibly, probably, or related for selected events, worst grade overall as reported for each patient. (**c**) Kaplan-Meier curves of OS according to the platinum agent used at the start of the treatment. (**d**) Kaplan-Meier curves of PFS according to the platinum agent used at the start of the treatment. There was no significant difference in OS or PFS for carboplatin vs. cisplatin-based chemotherapy (log rank p = 0.18 and p = 0.4 respectively). All p values are two-sided. Source data

The study enrolled the full planned cohort of 55 patients after initial safety analysis. The most commonly reported treatment-emergent adverse events (TEAEs) were mostly of low grade and included fatigue (67%), nausea (56%) and anemia (56%) (Extended Data Fig. 3). Grade 3 or higher TEAEs occurred in 65.5% of patients and included anemia (20%), hyponatremia (9%), fatigue (7%), leucopenia (5%), thrombocytopenia (5%) and hypertension (5%); all other grade 3 or higher TEAEs occurred in fewer than 5% of patients. No grade 5 TEAEs were observed. There were also no unexpected adverse events of special interest (defined as adverse events with a potential immune-mediated mechanism), and those that did occur were grade 2 or lower (Extended Data Fig. 3). In terms of treatment received, 29 (53%) patients received cisplatin-based chemotherapy from the start of treatment, whereas the rest began with carboplatin, and an additional seven patients in the cisplatin group switched to carboplatin during concurrent treatment. No statistically significant differences in PFS or OS were observed based on which platinum agent was received (Extended Data Fig. 3). All patients who enrolled in the study received at least one cycle of durvalumab with chemotherapy, and 87.3% (48/55) completed six cycles of concurrent treatment. Ten (18.2%) patients with epithelioid MPM completed the 1-year study treatment, and five (9.1%) patients discontinued the study treatment owing to toxicity. The median dose intensity (of cycles received) was 100% for cisplatin (range, 75–100), 100% for pemetrexed (range, 75–102) and 100% for durvalumab. Two (7%) of 29 patients who received cisplatin received a dose reduction of cisplatin owing to toxicity, and four (12%) of 33 who received carboplatin received a dose reduction of carboplatin owing to toxicity. One (2%) patient received a dose reduction of pemetrexed owing to toxicity, whereas no patients received a dose reduction of durvalumab owing to toxicity (Extended Data Fig. 1).

### Genomic and immunologic exploratory analyses

**Fig. 2:**
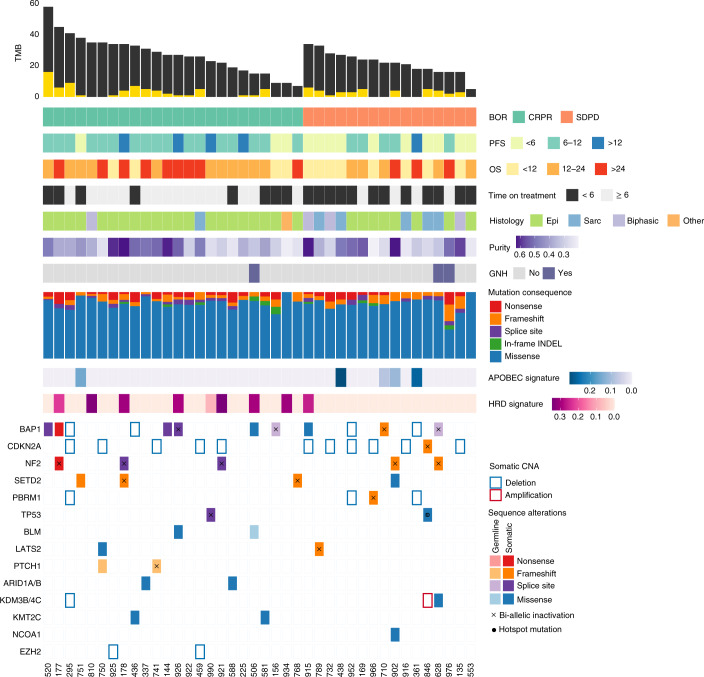
Genomic landscape of chemo-immunotherapy-treated mesotheliomas. MPMs of patients with a radiographic response harbored a higher number of non-synonymous missense sequence mutations (*n* = 40 MPM tumors; on average, 23 versus 18 mutations per exome for responding and non-responding tumors, respectively; Mann–Whitney *P* = 0.086). Epithelioid MPMs responding to therapy harbored a higher number of clonal missense mutations (*n* = 29 tumors; Mann–Whitney *P* = 0.051 and *P* = 0.025 for missense mutation load and clonal mutations, respectively); the numbers of subclonal mutations are shown as yellow inserts. Recurring inactivating alterations in *BAP1*, *CDKN2A*, *NF2*, *TP53*, *SETD2* and *PBRM1* did not differentially cluster with regard to therapeutic responses. Similarly, somatic *BAP1* sequence alterations and *CDKN2A* homozygous deletions were detected in 32.5% (13 of 40) and 30% (12 of 40) of MPMs, without a notable enrichment with respect to therapeutic response. Specific genotypes were associated with exceptional therapeutic outcome (PFS ≥12 months and/or OS ≥24 months): patient 178 harbored tumor biallellic inactivation of *NF2* and the histone methyltransferase *SETD2;* patient 926 harbored tumor biallellic inactivation of *BAP1*; and patient 361 harbored tumor homozygous deletions in *BAP1* and in the SWI/SNF nucleosome remodeling gene *PBRM1*. An enrichment in mutations in chromatin-regulating genes was observed for patients achieving an OS of 12 or more months (Fisher’s exact *P* = 0.063). We identified a higher contribution of an HRD mutation signature in responsive tumors (*n* = 40 patients; average HRD contribution of 9.1% versus 1.6% in responding and non-responding tumors, respectively; Mann–Whitney *P* = 0.043). Conversely, an APOBEC mutation signature was found to be more enriched in non-responding MPM and epithelioid MPM tumors (Mann–Whitney *P* = 0.058 and *P* = 0.031, respectively). Mutations were characterized by consequence (missense, frameshift, nonsense and splice site) and recurrence (hotspots, depicted as solid circles), and loss of the wild-type allele was considered in case of truncating mutations (biallellic inactivation, marked with an ‘x’). Tumor samples from patients 329, 351, 629 and 923 were excluded from analyses of somatic alterations owing to tumor tissue quality and are not shown here; these patients were included in the germline analyses, with patient 629 harboring a deleterious mutation in *BAP1*. BOR, best overall response; Epi, epithelioid; Sarc, sarcomatoid. Source data

**Extended Data Fig. 4:**
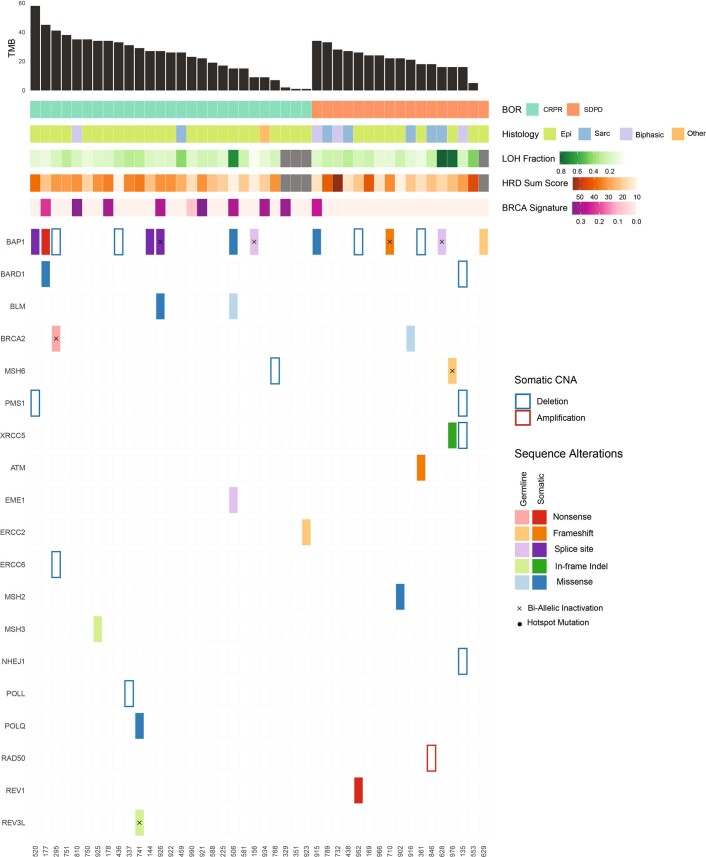
Distribution of mutations in genes involved in DNA damage repair. We investigated co-occurrence of mutations in DNA damage repair (DDR) genes involved in base excision repair, DNA damage sensoring, the Fanconi anemia pathway, homologous recombination, mismatch repair, nucleotide excision repair, non-homologous end joining and translesion DNA synthesis. Mutations were characterized by consequence (missense, frameshift, nonsense, splice site, in-frame) and recurrence (hotspots) and loss of the wild type allele was considered in case of truncating mutations (biallellic inactivation, marked with an ‘x’). There were no significant differences in somatic genomic alterations in the DDR gene set between tumors from responders and non-responders. When confirmed pathogenic germline mutations in DDR genes were considered, 5 patients harboring such alterations (629-BAP1 c.1717del, 295-BRCA2 c.6730 A>T, 628-BAP1 c.581-1 G > T, 976-MSH6 c.3261dup and 506-BLM c.968 A>G) had a longer overall survival as shown in Fig. 2. Abbreviations: TMB; tumor mutation burden, BOR; best overall response, CR; complete response, PR; partial response, SD; stable disease, PD; progressive disease, LOH; loss of heterozygosity, HRD; homologous recombination defect, CNA; copy number aberration.

**Extended Data Fig. 5:**
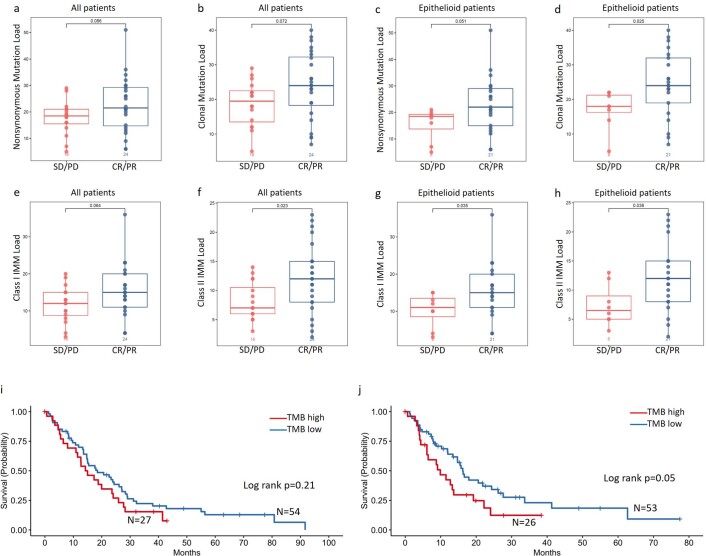
Immunogenic mutation load may be linked with functional neopeptide-specific anti-tumor immune response and therapeutic response. (**a**) Patients with MPM harboring a high non synonymous mutation load were more likely to achieve a radiographic response to chemo-immunotherapy (n = 40 MPM tumors, Mann Whitney p = 0.086). (**b**) MPMs responsive to chemo-immunotherapy also harbored a more clonal TMB (n = 40 MPM tumors, Mann Whitney p = 0.076). (**c**-**d**) These findings were more prominent in the epithelioid MPM group (n = 29 epithelioid MPM tumors, Mann Whitney p = 0.051 for non synonymous mutation load and Mann Whitney p = 0.025 for clonal mutation burden). (**e**,**f**) Immunogenic mutations associated with HLA class I and II neoantigens (Methods) better distinguished responders from non responders (n = 40 MPM tumors, Mann Whitney p = 0.064 for class I IMMs and Mann Whitney p = 0.023 for class II IMMs). (**g**,**h**) These findings were more pronounced for patients with epithelioid MPM (n = 29 epithelioid MPM tumors, Mann Whitney p = 0.035 for class I IMMs and Mann Whitney p = 0.038 for class II IMMs). The center line in the boxplots represents the median, the upper limit of the boxplots represents the third quantile (75th percentile), the lower limit of the boxplots represents the first quantile (25th percentile), the upper whiskers is the maximum value of the data that is within 1.5 times the interquartile range over the 75th percentile, and the lower whisker is the minimum value of the data that is within 1.5 times the interquartile range under the 25th percentile. (**i**) Survival analyses did not reveal any significant differences in overall survival between MPM tumors in the TCGA cohort with high TMB vs. those harboring a low TMB (log rank p = 0.21). (**j**) Analyses of progression-free survival revealed a trend towards longer PFS for patients with MPM tumors in the TCGA cohort harboring a low TMB (log rank p = 0.05). The TMB-high and low groups were defined using the 2nd quartile of TMB in the TCGA mesothelioma cohort, which corresponds to a non-synonymous mutation burden ≥33 alterations/exome. All p values are two-sided. Abbreviations: IMM; immunogenic mutation, CR; complete response, PR; partial response, SD; stable disease, PD; progressive disease.

**Extended Data Fig. 6:**
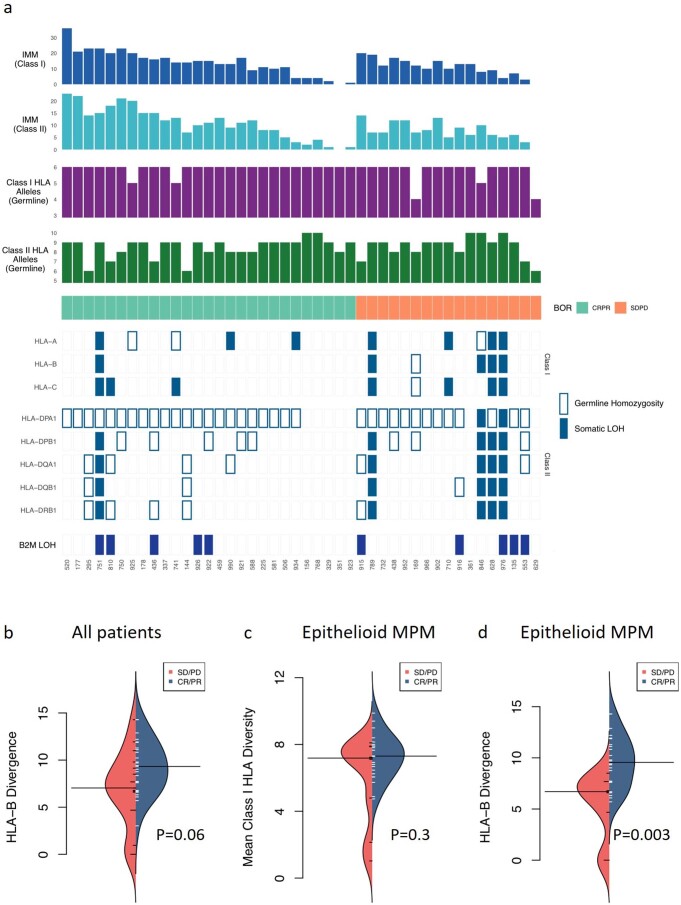
Germline and tumor HLA class I and II genetic variation. (**a**) Tumors from responders harbored a higher load of HLA class I and II immunogenic mutations (Methods, Mann Whitney p = 0.064 and 0.023 respectively) especially in the epithelioid MPM group (Mann-Whitney p = 0.035 and p = 0.038 respectively). There were no differences in the degree of germline homozygosity found between responders and non-responders. HLA class I and II germline zygosity and somatic LOH events were combined to calculate the unique number of HLA class I and class II alleles on cancer cells, which did not differ between responders and non responders. Similarly, there was no evidence of biallellic inactivation of β2-microglobulin or differential abundance of β2-microglobulin LOH events with respect to therapeutic response. (**b**) When sequence divergence of the allele’s peptide-binding domains was computed for HLA class I alleles, patients with high pairwise divergence between HLA alleles and in particular in HLA-B achieved a radiographic response (Mann Whitney p = 0.06), suggesting that in high HLA divergence may be linked with an increased diversity of the neopeptide repertoire presented and reflective of a more potent-antitumor immune response. (**c**) While there were no differences in mean HLA class I divergence for patients with epithelioid MPM (Mann Whitney p = 0.3), (**d**) a higher HLA-B divergence was linked with radiographic response (Mann Whitney p = 0.003). All p values are two-sided and unadjusted for multiple comparisons. Abbreviations: IMM; immunogenic mutation, B2M; β2-microglobulin, LOH; loss of heterozygosity.

As previously shown^17^, MPMs in this cohort harbored a low TMB, with some tumors harboring a higher than expected TMB in the setting of mutations in DNA damage repair genes (Fig. 2, Extended Data Fig. 4 and Supplementary Tables 1–3). Despite the notion that TMB might not predict response to immunotherapy for MPM, given the low mutation burden, we found that tumors from patients with a radiographic response to chemo-immunotherapy had a higher non-synonymous missense mutation burden and a more clonal mutation repertoire than non-responding tumors (*P* = 0.086 and *P* = 0.072, respectively; Extended Data Fig. 5 and Supplementary Table 4), especially in the epithelioid group (*P* = 0.051 and *P* = 0.025, respectively; Extended Data Fig. 5 and Supplementary Table 4). Consistent with these findings, an APOBEC mutational signature, reflective of subclonal mutagenesis, was found to be enriched in non-responding epithelioid tumors (*P* = 0.031; Fig.2 and Supplementary Table 4). To place these findings in context with respect to the potential role of TMB in predicting response to standard-of-care treatment versus chemo-immunotherapy, we analyzed tumor WES data from an independent cohort of 82 MPM tumors from The Cancer Genome Atlas (TCGA), which pre-dated the era of immune checkpoint blockade. In the TCGA MPM cohort, TMB-low tumors (TMB less than or equal to the second tertile in the cohort; Methods) showed a non-significant trend toward a longer PFS, suggesting that the relationship between high TMB and clinical response seen in the PrE0505 cohort might be driven by durvalumab (Methods and Extended Data Fig. 6). In the PrE0505 cohort, a trend toward an enrichment in mutations in chromatin-regulating genes was observed for patients achieving an OS of 12 or more months (*P* = 0.063).

**Extended Data Fig. 7:**
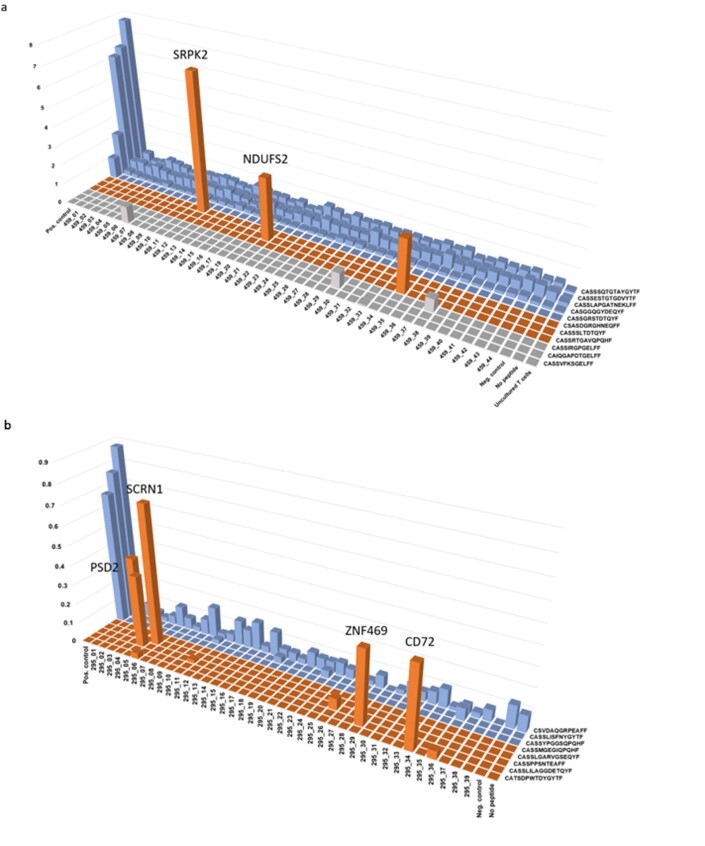
TCR clonotypic expansions to neopeptides derived from immunogenic mutations. (**a**) We used the MANAFEST assay, which detects memory T cell responses and identifies antigen-specific T cell receptors to test neoantigen-specific TCR reactivity. Neopeptides derived from class I immunogenic mutations were tested in vitro in autologous T cell cultures from peripheral blood of patient 459 that achieved an overall survival of 32.88 months after chemo-immunotherapy. Neopeptide-specific TCR clonotypic expansions were identified post stimulation with peptides derived from the SRPK2 p.C234Y and NDUFS2 p.V412L immunogenic mutations (orange bars, y axis denotes TCR productive frequencies of CD8+ T cells after the 10-day culture). (**b**) Neontigen-specific TCR clonotypic expansions were noted in autologous CD8+ T cell cultures from the peripheral blood for patient 295 that achieved an overall survival of 21.85 months and remained event-free at the time of data lock. TCR clonotypic expansions for peptides derived from the immunogenic mutations SCRN1 p.V334A, PSD2 p.C307Y, ZNF469 p.P3471S and CD72 p.T71A (orange bars, y axis denotes TCR productive frequencies of CD8+ T cells after the 10-day culture). Peptide pools of known HLA class I-restricted viral antigens (Methods) were utilized as positive controls and are shown in blue bars for both cases.

**Extended Data Fig. 8:**
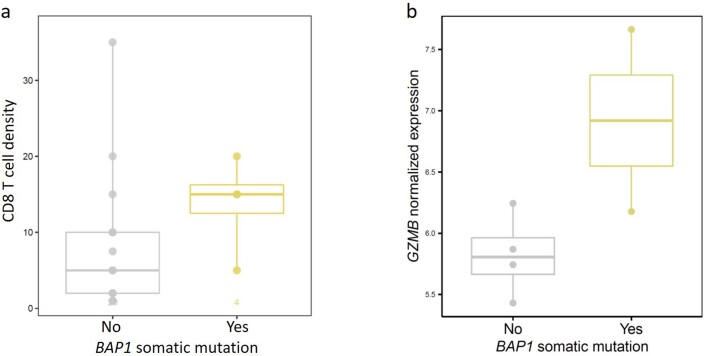
Differences in CD8+T cell infiltration in *BAP1* mutant tumors. (**a**) Epithelioid MPMs harboring somatic *BAP1* inactivating mutations had a higher intratumoral CD8+ T cell infiltration (n = 33 epithelioid MPM tumors, Mann Whitney p = 0.036). (**b**) RNA sequencing of tumor samples with adequate tissue (n = 6, 4 *BAP1* wild type and 2 *BAP1* mutant) revealed a higher expression level for GranzymeB in *BAP1* mutant tumors (n = 6 MPM tumors, Mann Whitney p = 0.0094). The center line in the boxplots represents the median, the upper limit of the boxplots represents the third quantile (75th percentile), the lower limit of the boxplots represents the first quantile (25th percentile), the upper whiskers is the maximum value of the data that is within 1.5 times the interquartile range over the 75th percentile, and the lower whisker is the minimum value of the data that is within 1.5 times the interquartile range under the 25th percentile. All p values are two-sided. Source data

We then evaluated non-synonymous sequence alterations associated with putatively immunogenic neoantigens (immunogenic mutations (IMMs); Methods and Supplementary Table 3) and found an enrichment of high major histocompatibility complex (MHC) class I IMM burden (*P* = 0.064) as well as a higher MHC class II IMM burden (*P* = 0.023) in responsive tumors (Extended Data Fig. 5 and Supplementary Table 4), especially for epithelioid MPM (*P* = 0.035 and *P* = 0.038, respectively). Interestingly, consistent with the HLA class I allele divergence hypothesis that points toward a more efficient tumor immune surveillance in the presence of HLA class I functional diversity^25^, maximal germline physiochemical sequence divergence at the HLA-B locus was associated with radiographic response, especially for epithelioid MPM (*P* = 0.06 and *P* = 0.003, respectively; Extended Data Fig. 7 and Supplementary Table 5). We subsequently tested autologous T cells for reactivity against IMM-derived neopeptides for two patients who achieved long-term clinical response (Methods). T cell receptor (TCR) clonotypic expansions for neoantigens derived from the *SRPK2* p.C234Y and *NDUFS2* p.V412L mutations were prominent for patient 459 with sarcomatoid MPM and an OS of 33 months (Extended Data Fig. 8). Similarly, neoantigen-specific TCR expansions were noted for the IMMs *SCRN1* p.V334A, *PSD2* p.C307Y, *ZNF469* p.P3471S and *CD72* p.T71A for patient 295 with epithelioid MPM and an OS of 21.85 months (patient remained event-free at the time of data lock; Extended Data Fig. 8).

**Fig. 3:**
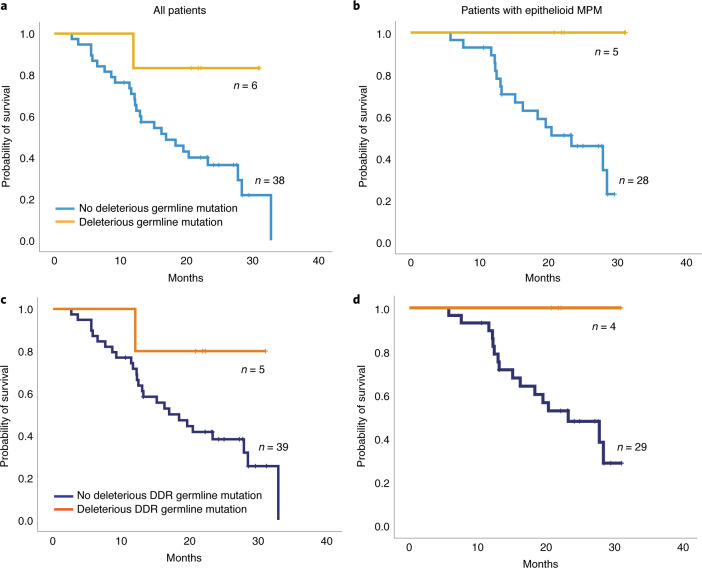
Effect of germline mutations in cancer susceptibility genes on outcome from combined immuno-chemotherapy. **a**, **b**, Patients harboring known deleterious germline mutations in mesothelioma-predisposing genes (Methods) had a longer OS (log-rank *P* = 0.05), especially in the epithelioid MPM group (log-rank *P* = 0.032). **c**, **d**, A focused analysis including deleterious germline mutations in *BAP1*, *BRCA2*, *MSH6* and *BLM*—all genes involved in DNA damage repair—showed the same trends toward a longer OS for patients harboring germline mutations in DDR genes (log-rank *P* = 0.12 for all patients and log-rank *P* = 0.082 for patients with epithelioid tumors). All *P* values are two sided. DDR; DNA damage repair.

**Extended Data Fig. 9:**
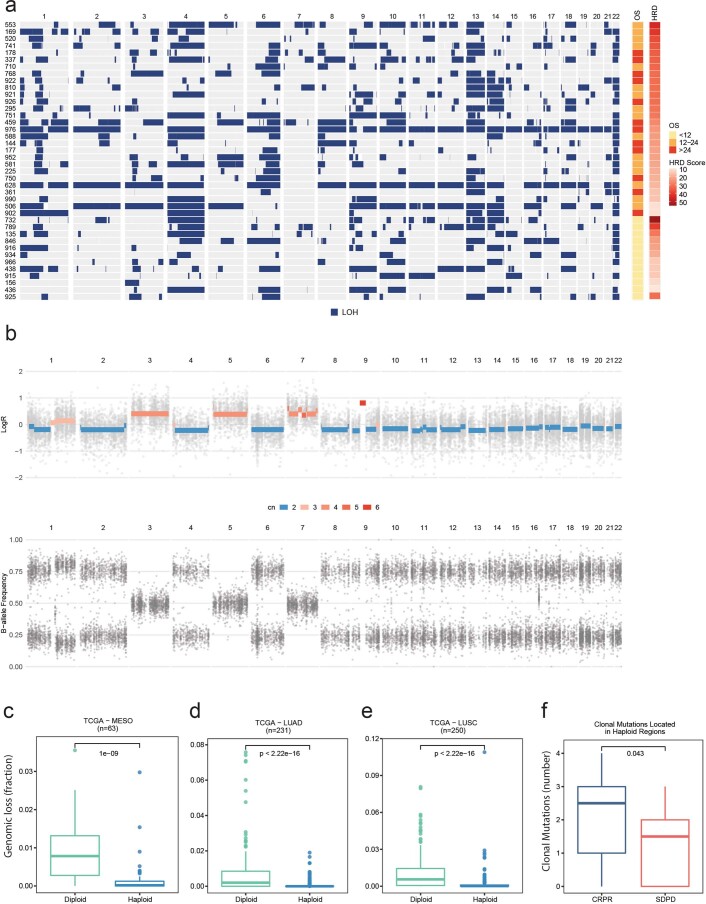
Genome-wide loss of heterozygosity and genome near haploidization. (**a**) MPM tumors exhibited various levels of genome-wide loss of heterozygosity. LOH in chromosomal arms 4p, 4q, 6q, 13q (containing the *BRCA2*, *ERCC5* and *LATS2* loci), 14q (containing the *FANCM*, *MLH3* and *XRCC3* loci), and 22q (containing the *CHECK2*, *NF2* and *XRCC6* loci) was observed at a significantly higher rate compared to background. In three tumors (cases 506, 628, 976), LOH was widespread throughout the genome consistent with the genome near haploidization phenotype. (**b**) Genome-wide copy number (top) and B-allele frequency (bottom) profiles of a tumor with GNH (976), depicting a hyper-diploid state and LOH in all chromosomes observed at normal copy number state. Abbreviations: LOH; loss of heterozygosity, GNH; genome near haploidization. (**c**-**e**) Comparison of the background rate of genomic loss in 1,086 mesothelioma and non-small cell lung cancer cases from TCGA showing that haploid regions of the genome consistently harbored a lower background rate of genomic loss compared to diploid regions (Mann Whitney p = 1e-09, p < 2.2e-16 and p < 2.2e-16 for mesothelioma, lung adenocarcinoma and lung squamous cell carcinomas respectively). (**f)** Patients in the PrE0505 study with a radiographic response harbored a higher number of clonal mutations within the haploid regions of their genomes (n = 40 MPM tumors, Mann Whitney p = 0.043). The center line in the boxplots represents the median, the upper limit of the boxplots represents the third quantile (75th percentile), the lower limit of the boxplots represents the first quantile (25th percentile), the upper whiskers is the maximum value of the data that is within 1.5 times the interquartile range over the 75th percentile, and the lower whisker is the minimum value of the data that is within 1.5 times the interquartile range under the 25th percentile. All p values are two-sided and unadjusted for multiple comparisons. Abbreviations: MESO; mesothelioma, LUAD; lung adenocarcinoma, LUSC; lung squamous cell carcinoma, CR; complete response, PR; partial response, SD; stable disease, PD; disease progression. Source data

Mesothelioma may arise in the context of germline mutations in cancer susceptibility genes, including *BAP1*, *MLH1*, *MLH3, BRCA1*, *BRCA2* and *BLM*^20,22^; however, the potential effect of germline MPM-predisposing mutations on response to chemo-immunotherapy has not been previously evaluated. Patients with pathogenic germline loss-of-function mutations in MPM susceptibility genes (Methods), predominantly those involved in DNA damage repair, had a significantly prolonged survival (log-rank *P* = 0.05 and *P* = 0.032 for all patients and epithelioid MPM, respectively; Fig. 3a–d). Interestingly, patients with sporadic *BAP1* mutant MPM harboring heterozygous somatic inactivating mutations did not have a better radiographic response or longer OS, potentially suggesting that immune surveillance mechanisms differ in the context of germline BAP1 deficiency^26,27^. As previously described^4,28^, patients with inactivating *BAP1* germline mutations were younger (*P* = 0.009)^29,30^, and tumors with *BAP1* biallellic inactivation were found to have an enriched DNA mismatch repair deficiency-related mutational signature (*P* = 0.003). Tumors harboring somatic *BAP1* inactivating mutations were noted to have a higher clonal TMB as well as a lower fraction of genome with loss of heterozygosity (LOH) (*P* = 0.033 and *P* = 0.018, respectively); notably a trend toward a lower degree of LOH was also observed for *BAP1* mutant mesotheliomas in the TCGA cohort. Interestingly, these findings were not corroborated in cases harboring germline inactivating *BAP1* mutations, highlighting the differences between germline and somatic genetic backgrounds. Furthermore, we found that epithelioid MPMs harboring somatic *BAP1* inactivating mutations had a higher intratumoral CD8^+^ T cell infiltration (Extended Data Fig. 9). Analyses of transcriptomic sequence data for six patients with available tissue (four *BAP1* wild-type and two *BAP1* mutant) revealed a higher expression level for granzyme B in *BAP1* mutant tumors, suggesting an active cytotoxic immune response in the microenvironment of *BAP1* mutant tumors (Extended Data Fig. 9).

**Fig. 4:**
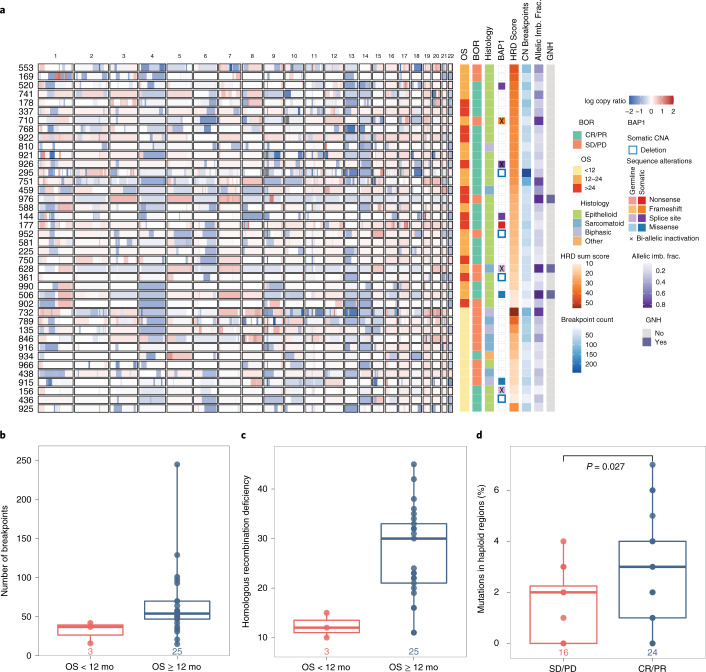
Large-scale copy number analyses. **a**, Genome-wide copy number analyses predominantly revealed genomic regions with copy number losses (shown in blue) and were used to determine the extent of copy number breakpoints and fraction of genome with complete allelic imbalance, reflecting genomic instability and tumor aneuploidy. The relative copy ratio (log copy ratio) values quantifying the abundance of each genomic region compared to the average genome ploidy are shown per chromosome after correction for tumor purity. Red and blue shades indicate copy gains and losses, respectively, whereas white marks indicate copy neutral regions. An HRD score was computed, taking into account telomeric allelic imbalance, LOH and large-scale state transitions. Three extreme cases of LOH were noted, with a copy number pattern that was suggestive of genome near-haploidization; these patients had an OS of 12 or more months after chemo-immunotherapy. **b**, **c**, A higher number of copy number breakpoints and a higher HRD score distinguished epithelioid MPM from patients with an OS of 12 or more months (*n* = 28 epithelioid MPM tumors; Mann–Whitney *P* = 0.0.05 and *P* = 0.014, respectively). **d**, Responding tumors harbored a higher number of mutations in single-copy regions of the genome, suggesting that these ‘difficult’ to eliminate alterations and associated neoantigens might be important drivers of the anti-tumor immune response (*n* = 40 MPM tumors; Mann–Whitney *P* = 0.027). The center line in the box plots represents the median; the upper limit of the box plots represents the third quantile (75th percentile); the lower limit of the box plots represents the first quantile (25th percentile); the upper whisker is the maximum value of the data that are within 1.5 times the interquartile range over the 75th percentile; and the lower whisker is the minimum value of the data that are within 1.5 times the interquartile range under the 25th percentile. All *P* values are two sided. Allelic imb. frac., fraction of genome with allelic imbalance; BOR, best overall response; CNA, copy number alteration. Source data

**Extended Data Fig. 10:**
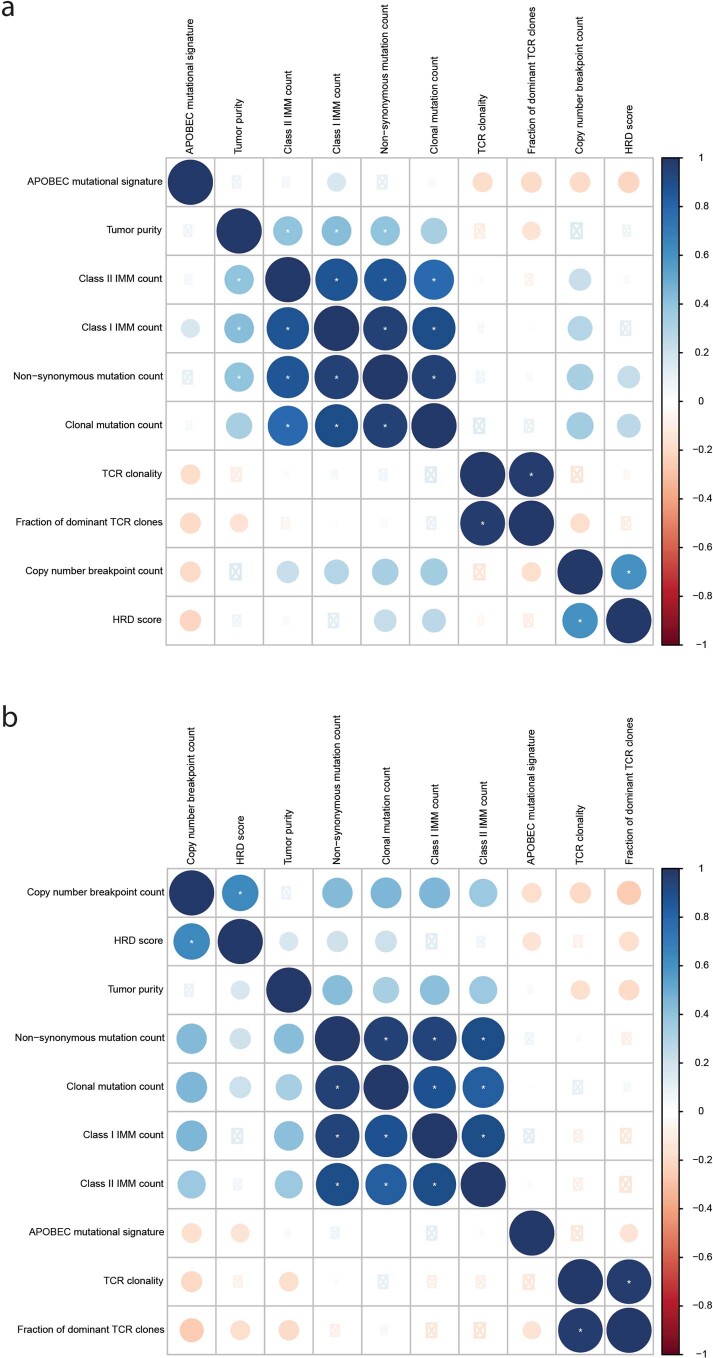
Correlations between genomic and molecular features. Non parametric correlations between sequence and structural genomic and TCR features for all patients (**a**) and patients with epithelioid mesothelioma (**b**). Correlations between tumor purity and non-synonymous missense mutation burden and associated neoantigens were noted for all patients but not for the epithelioid mesothelioma group. An asterix indicates a p value less than 0.05.

Genomic instability and, particularly, large-scale copy number losses are hallmarks of MPM^18,29^, and our large-scale copy number analyses revealed recurrent chromosomal arm losses or LOH of 4p, 4q, 6q, 9p, 10q, 13q, 14q, 18q and 22q as well as LOH/deletion of 3p21.1, where *BAP1* lies (Fig. 4a, Supplementary Tables 6 and 7 and Extended Data Fig. 10). Regions of recurrent copy loss contained the *LATS1* (6q), *CDKN2A* (9p), *LATS2* (13q) and *NF2* (22q) loci. We found an enrichment for LOH of chromosomal arm 1p and hemizygous loss of chromosomal arm 6q in tumors that did not achieve a radiographic response (two of 24 in the responder versus six of 16 in the non-responder group, *P* = 0.04; Fig. 4a). Interestingly, loss of chromosomal arm 6q, which contains the mesothelioma driver genes *LATS1*, *REV3L* and *SHPRH*, was more prominent in epithelioid tumors without treatment response (two of 21 in the responder group versus four of eight in the non-responder group, *P* = 0.033). As DNA breaks occur frequently in mesothelioma^17,19^, we quantified chromosomal instability by estimating the number of copy number breakpoints across the genome (Methods) and found a higher content of genome-wide breakpoints in epithelioid MPM of patients with an OS of 12 or more months (*P* = 0.053; Fig. 4b and Supplementary Table 4). Analysis of breakpoints of the TCGA mesothelioma cohort failed to identify such an association with OS (univariate Cox proportional hazards regression analysis, HR = 1.36, *P* = 0.23). Furthermore, we computed a composite homologous recombination deficiency (HRD) score, incorporating telomeric allelic imbalance, LOH and large-scale state transitions and found that a higher HRD score defined epithelioid tumors from patients with long-term survival (*P* = 0.014; Fig. 4c and Supplementary Table 4); by contrast, no association was observed between a higher HRD score and outcome in the TCGA mesothelioma cohort (either as a continuous variable (HR = 1.02, *P* = 0.14) or a binarized variable (HR = 1.58, *P* = 0.18), using univariate Cox proportional hazards regression analysis). Consistent with these findings, a mutational signature of DNA double-strand break repair deficiency was more enriched in tumors of patients with a radiographic CR or PR (*P* = 0.043; Fig. 2 and Supplementary Table 4). Three cases showed genomic near haploidization, which is a phenomenon where cells lose one copy of nearly all chromosomes followed by duplication of the remaining chromosomes, and, interestingly, all of these patients had an OS of 12 or more months (Fig. 4a and Extended Data Fig. 10). It is plausible that mutations and associated neoantigens contained in regions of the genome with a single copy per cancer cell cannot be eliminated under the selective pressure of therapy and, therefore, mediate sustained neoantigen-driven immune responses and long-term clinical benefit. Consistent with this notion, we discovered a higher number of sequence alterations contained in single-copy regions of the genome in tumors from responders compared to non-responders (*P* = 0.027; Fig. 4d); notably, the overwhelming majority of these were clonal (71 of 93, 76%; Supplementary Table 8). To further support these findings, we investigated the background rate of loss in regions of the genome with a single copy per cell (haploid regions) versus two copies per cell (euploid regions) and analyzed somatic copy number profiles of 1,086 mesothelioma and non-small-cell lung cancer tumors from TCGA (Methods). These analyses revealed that the rate of loss in haploid regions was consistently lower than that of euploid regions (Extended Data Fig. 9), supporting the notion that mutations contained in these regions are difficult to eliminate and might drive a sustained anti-tumor immune response.

**Fig. 5:**
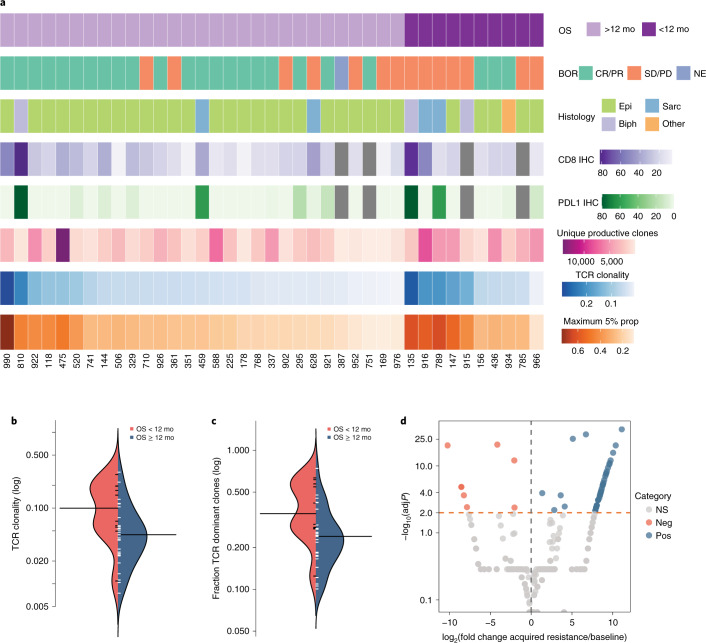
Baseline TCR repertoire characteristics and dynamic changes at the time of therapeutic resistance. **a**, The intratumoral T cell repertoire was interrogated by TCR Vβ sequencing; clonality of the TCR repertoire was computed; and the representation of dominant clones (Methods) as a proportion of the whole TCR repertoire was determined. CD8^+^ T cell density and PD-L1 tumor proportion scores for each evaluable case are shown (missing cases are shown in gray). **b**, These analyses revealed a less clonal TCR repertoire in tumors from patients achieving an OS of 12 or more months (Mann–Whitney *P* = 0.018). **c**, A higher representation of dominant clones was also detected in tumors from patients with a shorter OS (Mann–Whitney *P* = 0.006). **d**, Differential abundance analyses of three cases with available tumor samples before therapy initiation (295, 459 and 926) and at the time of acquired resistance revealed TCR clonotypic expansions (labeled as significant positive) and regressions (labeled as significant negative) as shown for patient 926, who had a PR and an OS of 27.8 months. Fold change of intra-tumoral TCR clones is plotted on the *x* axis (log scale), and the adjusted corresponding Mann–Whitney *P* value is shown on the *y* axis (−log scale) of the volcano plot. All *P* values are two sided. Biph; biphasic; BOR, best overall response; Epi, epithelioid; IHC, immunohistochemistry; NE, non-evaluable; Neg, significantly negative (regressing TCR clones); NS, not significant; Pos, significantly positive (expanding TCR clones); Sarc, sarcomatoid. Source data

As reported previously^15^, we did not observe any association between radiographic responses, PFS or OS and PD-L1 expression on tumor cells. In looking at the tumor microenvironment of MPM, the composition of the pre-existing intra-tumoral TCR repertoire tied into the genomic footprint of MPM has not been previously investigated in the context of chemo-immunotherapy. Baseline tumors of patients with an OS of 12 or more months harbored a more diverse TCR repertoire in contrast to tumors of patients with shorter OS, which showed a higher TCR repertoire clonality and a higher proportion of high-frequency TCR clones (*P* = 0.018 and *P* = 0.006, respectively; Fig. 5a–c and Supplementary Tables 4 and 9). We investigated the reshaping of the intra-tumoral T cell repertoire for three patients (295, 459 and 926) who had long-term therapeutic responses but eventually developed acquired resistance, by serially sampling tumors before therapy and at the time of acquired resistance. Interestingly, at the time of acquired resistance, significant reshaping signified by TCR clonotypic expansions and regressions was noted, such that the tumor-infiltrating lymphocyte (TIL) repertoire from all three cases was significantly more clonal (Fig. 5d). These findings potentially suggest that an effective anti-tumor immune response is mediated by a polyclonal T cell repertoire, and dependency on fewer TCR effector cells might not be sufficient to mount an effective anti-tumor immune response.

## Discussion

The PrE0505 trial delivered promising rates of response, PFS and OS for patients who received durvalumab with standard chemotherapy as first-line therapy for unresectable MPM. Treatment was well tolerated, and there were low rates of immune-mediated toxicity. The median OS of 20.4 months in the PrE0505 trial is encouraging in the context of several recent phase 2 and 3 clinical trials that enrolled a similarly representative population of treatment-naive patients^7–9,16^. Allied to recent results from the DREAM study, these data launched the ongoing phase 3 PrE0506/DREAM3R trial (NCT04334759), which compares durvalumab with chemotherapy to chemotherapy alone^15^. Notably, the survival for patients with epithelioid MPM in the PrE0505 trial exceeded 2 years, and several patients with epithelioid MPM continue to be free from tumor progression at the time of this publication. This potential benefit from chemo-immunotherapy for epithelioid MPM is in contrast to the recent CheckMate 743 trial that reported a striking survival advantage favoring ipilimumab–nivolumab over chemotherapy for patients with non-epithelioid histology (18.1 versus 8.8 months); however, no significant survival difference was observed between the two treatment arms for patients with epithelioid MPM (18.7 versus 16.5 months)^16^. Given the known chemosensitivity of epithelioid MPM and the relative chemo-resistance of non-epithelioid MPM, it is possible that chemo-immunotherapy confers a synergistic advantage, particularly for patients with epithelioid MPM. As both DREAM and PrE0505 trials mandated that patients conclude durvalumab treatment after one full year of treatment, it is conceivable that some patients would derive additional benefit from maintenance therapy until disease progression, although data on this point are conflicting across tumor types^30^. The investigational arm of the ongoing phase 3 PrE0506/DREAM3R trial includes treatment with maintenance durvalumab until confirmed disease progression, thus addressing this potential concern.

The clinical efficacy of chemo-immunotherapy shown in the PrE0505 trial challenged the common paradigm of immunotherapy-responsive tumors, as MPM harbors a low non-synonymous TMB that conceptually might limit the number of presented immunogenic neoantigens. Although tumors with TMB in the lower end of the spectrum are historically thought to have TMB-independent mechanisms of response to immunotherapy^19^, we discovered that a higher immunogenic mutation load distinguished responding tumors, particularly in the epithelioid MPM group. Notably, these findings were not corroborated in MPM treated with standard-of-care therapies, suggesting an association with durvalumab. Clonal TMB represents a dominant tumor-intrinsic determinant of clinical response to immunotherapy^31^, which is consistent with our findings of clonal TMB predicting radiographic response in epithelioid MPM. Similarly, a high subclonal mutation burden, in part mediated by abnormal activity of the APOBEC enzymes, might enable tumor immune escape^32,33^. We indeed discovered an inverse association between an APOBEC mutational signature and response to chemo-immunotherapy in epithelioid MPM in the PrE0505 cohort. To substantiate these findings, we pulsed autologous T cells with peptides derived from immunogenic mutations and identified neoantigen-specific TCR expansions in vitro, suggesting that robust neoantigen-specific responses were linked with favorable clinical outcome.

Consistent with the notion that MPM is driven by inactivating mutations in tumor suppressor genes^17,18,29,34,35^, we identified recurring inactivating largely non-overlapping genomic alterations in *BAP1*, *CDKN2A, NF2*, *SETD2*, *PBRM1* and *TP53* independent of therapeutic response. Although we did not find an enrichment in alterations of any single gene in tumors from patients with differential responses to chemo-immunotherapy, a trend toward an enrichment in somatic mutations in chromatin-regulating genes was noted in tumors from patients achieving an OS of 12 or more months; these alterations might mediate transcriptional changes of genes involved in immune-related signaling pathways^36^ or might be linked with a genomic instability phenotype that can predispose to response to immunotherapy^37^.

Germline genetic susceptibility has been established as a seminal event in MPM tumorigenesis, mostly involving tumor suppressor genes in DNA repair mechanisms^20,22,29,38^. The frequency of 32% for *BAP1* genomic alterations in the PrE0505 cohort is in line with these previously reported analyses^17,29,39^. Presence of germline *BAP1* mutations has been linked with a longer 5-year survival, suggesting a less aggressive phenotype of MPM arising in the context of a *BAP1* cancer syndrome^28,39,40^. The underlying etiology of this phenomenon remains unclear, with one potential explanation being that the tumor microenvironment of BAP1 null tumors is more inflammatory^27^. Although we did not find a prolonged survival for patients with somatic *BAP1* mutations, *BAP1* mutant tumors were found to have a higher degree of CD8^+^ T cell infiltration. Notably, patients harboring deleterious germline mutations in MPM-predisposing genes, including, but not limited to, genes involved in DNA homologous recombination, achieved significantly longer PFS and OS with chemo-immunotherapy. Inherited defects in homologous recombination repair, resulting in microdeletions and DNA breaks, might be linked with longer OS after platinum chemotherapy^20,28^ as well as affect adaptive and innate immunity, ultimately potentiating response to immune checkpoint blockade^41,42^. Our findings suggest that germline genotypes might affect clinical outcomes after chemo-immunotherapy, and germline testing should be considered for clinical decision-making for patients with mesothelioma.

Notably, we found that DNA repair deficiency, and defective homologous recombination in particular, determined by both sequence mutational spectra and genome-wide copy number analyses, were hallmarks of responding tumors, especially for epithelioid MPM. Overall, responding epithelioid tumors harbored a higher content of genome-wide copy number breakpoints, suggesting that genomic instability affects therapeutic efficacy for chemo-immunotherapy. Although we did not detect any evidence of oscillating copy number changes within any given chromosome indicative of chromothripsis in the PrE0505 cohort, there were three cases with extensive genome-wide LOH, a phenomenon called genome near-haploidization (GNH), which has been previously reported in five MPM cases^17^. Interestingly, two of the tumors with GNH in our cohort harbored *BAP1* mutations, and all patients achieved an OS longer than 12 months. As GNH-harboring MPM might comprise a novel molecular subtype of MPM with distinctive clinical behavior^17^, our findings suggest that these unique genomic features might be linked to favorable response to chemo-immunotherapy. Conceptually, immunogenic mutations residing in genomic loci that undergo haploidization cannot be lost under the selective pressure of immunotherapy^43^ and, therefore, might drive a sustained anti-tumor immune response. Consistent with this hypothesis, we discovered that tumors that harbored a higher number of sequence alterations in single-copy regions of the genome responded to combined chemo-immunotherapy.

The density of the CD8^+^ T cell infiltrate has been associated with effective anti-tumor immune responses^44,45^; however, in the PrE0505 cohort, neither CD8^+^ T cell infiltration nor PD-L1 protein expression predicted response to chemo-immunotherapy. Notably, the tumor microenvironment of responding tumors contained a less clonal TCR repertoire that become more polarized at the time of acquired resistance. In contrast to the notion that anti-tumor immune responses in the context of immunotherapy are driven by a clonal T cell repertoire in TMB-high tumors such as melanoma^44^ or non-small-cell lung cancer^46^, our findings suggest that maximal immune cell repertoire diversity is required to mount an effective anti-tumor immune response in MPM. Consistent with this notion, a higher TCR diversity has been previously shown to correlate with improved outcome in bladder cancer, colorectal cancer, hepatocellular carcinoma and uterine cancer^47^.

Our study was limited by its small sample size and absence of a non-durvalumab control arm. To interpret our molecular findings with respect to response to chemo-immunotherapy compared to standard-of-care therapy alone, we performed genomic analyses of an independent cohort of 82 mesotheliomas obtained from the TCGA registry. This cohort did not include patients treated with chemo-immunotherapy or immunotherapy, and analyses of the TCGA mesothelioma cohort suggested that the genomic features of response in the PrE0505 trial were driven by durvalumab. Notably, definitively assessing the predictive versus prognostic nature of our findings is needed and planned within the ongoing phase 3 randomized DREAM3R/PrE0506 trial. Furthermore, although the control survival was chosen based on the historical control that led to the approval of pemetrexed with cisplatin^5^, recent randomized data have shown both shorter and longer survival for the pemetrexed–cisplatin combination^8,9^; it is, therefore, possible that control assumptions might have underestimated the expected survival with chemotherapy alone.

In summary, we report the favorable clinical efficacy of the PrE0505 study of chemo-immunotherapy for unresectable MPM with in-depth molecular and functional analyses, which provide an understanding of the complex genomic and immune cell features of response to combined chemo-immunotherapy with potential broad clinical implications.

## Methods

### Trial design, endpoints, oversight and samples analyzed

The first patient enrollment date was 12 June 2017; the last patient enrollment date 21 June 2018. A detailed outline of the numbers of patients available for analyses is shown in the CONSORT diagram in Extended Data Fig. 1. Owing to biospecimen availability and biospecimen quality, germline genomic data were evaluable for 44 patients, and somatic genomic data were evaluable for 40 patients (Supplementary Table 1 and Extended Data Fig. 1). A data safety monitoring board reviewed the study twice a year, and patient consent was provided for sample collection.

The full list of the inclusion and exclusion criteria is shown below:

- Histologically and/or cytologically confirmed MPM
- Unresectable disease (defined as the participant not being a candidate for curative surgery)
- Measurable disease, defined as at least one lesion (measurable) that can be accurately assessed at baseline by computed tomography or magnetic resonance imaging and is suitable for repeated assessment (modified RECIST for pleural mesothelioma)
- Available unstained archived tumor tissue sample in sufficient quantity to allow for analyses. At least 15 unstained slides or a tumor block (preferred). Note: A fine needle aspiration sample is not sufficient to make the patient eligible for enrollment. Given the complexity of mesothelioma pathological diagnosis and that these will be newly diagnosed patients, it is expected that they will have a core needle biopsy or surgical tumor biopsy as part of their initial diagnostic workup.
- Age ≥ 18 years
- ECOG performance status of 0 or 1
- Ability to understand and willingness to sign institutional review board-approved informed consent
- Willingness to provide archived tumor tissue and blood samples for research
- Adequate organ function as measured by the following criteria, obtained at most 2 weeks before registration:
  - Absolute neutrophil count ≥1,500 per mm³
  - Hemoglobin >9.0 g dl^−1^
  - Platelets >100,000 per mm^3^
  - Serum creatinine clearance >60 ml min^−1^ by the Cockcroft–Gault formula or by 24-h urine collection for determination of creatinine clearance. Note: Patients with a creatinine clearance ≥45 ml min^−1^ but ≤60 ml min^−1^ may be considered for enrollment provided they fulfill all other eligibility criteria. These patients will receive pemetrexed carboplatin concurrent with durvalumab during the combination phase of treatment. Patients with a creatinine clearance <45 ml min^−1^ should not be enrolled.
  - Albumin ≥2.8 g dl^−1^
  - Total bilirubin ≤1.5× the upper limit of normal (ULN)
  - Aspartate aminotransferase/alanine aminotransferas ≤2.5× ULN (≤5× ULN in patients with liver metastases)
- Women must either be of non-reproductive potential or have a negative serum pregnancy test upon study entry.
- Women must not be pregnant or breastfeeding.
- Patients are willing and able to comply with the protocol for the duration of the study, including undergoing treatment and scheduled visits and examinations, including follow-up.
- Patients must not have involvement in the planning and/or conduct of the study and no previous enrollment in the present study.
- Patients must not have participated in another clinical study with an investigational product during the last 4 weeks.
- Patients must not have received any prior systemic therapy (chemotherapy, immunotherapy, endocrine therapy, targeted therapy, biologic therapy, tumor embolization, monoclonal antibodies or other investigational agent) for mesothelioma.
- No previous treatment with a PD1 or PD-L1 inhibitor, including durvalumab or any other agent targeting immune checkpoints
- Patients must not have non-pleural mesothelioma—for example, mesothelioma arising in the peritoneum, tunica vaginalis or any serosal surface other than the pleura.
- Patients must not have an active second malignancy other than non-melanoma skin cancer or cervical carcinoma in situ.
- Patients must not have a mean QT interval corrected for heart rate (QTc) ≥470 ms calculated from three electrocardiograms using Frediricia’s correction.
- Patients must not have symptomatic or uncontrolled brain metastases requiring concurrent treatment, inclusive of, but not limited to, surgery, radiation and/or corticosteroids (prednisone >10 mg or equivalent). Surgery, radiation and/or corticosteroids (any dose >10 mg of prednisone equivalent) must have been completed 2 or more weeks before registration.
- Patients must not have uncontrolled seizures.
- Patients must not have current or prior use of immunosuppressive medication within 28 d before the first dose of durvalumab, with the exceptions of intranasal and inhaled corticosteroids or systemic corticosteroids at physiological doses, which are not to exceed 10 mg per day of prednisone or an equivalent corticosteroid. Standard steroid premedication given before chemotherapy or as prophylaxis for imaging contrast allergy should not be counted for this criterion.
- No active or prior documented autoimmune or inflammatory disorders (including inflammatory bowel disease, diverticulitis with the exception of diverticulosis, celiac disease, irritable bowel disease and Wegner syndrome) within the past 2 years. Patients with vitiligo, alopecia, Grave’s disease or psoriasis not requiring systemic treatment (within the past 3 years) are not excluded.
- No history of primary immunodeficiency
- No history of allogeneic organ transplant
- No history of hypersensitivity to durvalumab, cisplatin, carboplatin, pemetrexed or any of their excipients
- No uncontrolled intercurrent illness including, but not limited to, ongoing or active infection, symptomatic congestive heart failure, uncontrolled hypertension, unstable angina pectoris, cardiac arrhythmia, active peptic ulcer disease or gastritis and active bleeding diatheses, as well as any patient known to have psychiatric illness/social situations that would limit compliance with study requirements or compromise the ability of the patient to give written informed consent.
- No active infection, including tuberculosis (clinical evaluation including physical examination findings, radiographic findings and positive purified protein derivative test), hepatitis B virus (HBV) (known positive HBV surface antigen (HBsAg) result) and hepatitis C or HIV (positive HIV 1/2 antibodies as defined by a positive ELISA test). Patients with a past or resolved HBV infection (defined as the presence of hepatitis B core antibody (anti-HBc) and absence of HBsAg) are eligible. Patients positive for hepatitis C virus (HCV) antibody are eligible only if polymerase chain reaction (PCR) is negative for HCV RNA. HIV testing is not required in absence of clinical suspicion.
- No known history of leptomeningeal carcinomatosis
- Patients must not have received live attenuated vaccination within 30 d before study entry or within 30 d of receiving durvalumab.
- Patients must not have any condition that, in the opinion of the investigator, would interfere with the evaluation of study treatment or interpretation of patient safety or study results.

Imaging was performed every 6 weeks during the concurrent phase of treatment and every 9 weeks during maintenance durvalumab. Best objective response was evaluated by RECIST version 1.1 criteria modified for mesothelioma. Toxicity was determined using Common Terminology Criteria for Adverse Events version 4.03.

Secondary objectives included safety and tolerability of durvalumab and durvalumab in combination with chemotherapy in patients with MPM; percentage of patients who were progression-free at 24 weeks from the time of registration (response coded based on modified RECIST 1.1 criteria for mesothelioma); PFS, measured from the time of study registration until radiologic progression, clinical progression or death; and best objective response rate with evaluation continued for up to 1 year (response coded based on modified RECIST version 1.1 criteria for mesothelioma). Exploratory objectives included assessment of tumor baseline PD-L1 expression, the genomic and neoantigen landscape of tumors, dynamics of circulating cell-free tumor DNA and other blood-based biomarkers.

### WES

#### TCGA mesothelioma cohort

We obtained matched tumor-normal exome sequencing data from 82 patients with MPM in TCGA (http://cancergenome.nih.gov), as outlined in TCGA publication guidelines (http://cancergenome.nih.gov/publications/publicationguidelines). WES-derived somatic mutation calls from the TCGA PanCancer Atlas MC3 project were retrieved from the National Cancer Institute (NCI) Genomic Data Commons (https://gdc.cancer.gov/about-data/publications/, mc3-2017). The MC3 mutation call set is the result of application of a uniform analysis pipeline, including a standardized set of six mutation callers and an array of automated filters to all the entire TCGA exome data^48^. TMB was calculated as the number of non-synonymous mutations detected by WES.

### Tissue sample characteristics and sample preparation

Formalin-fixed, paraffin-embedded (FFPE) tumor tissue and matched peripheral blood were collected before therapy initiation. DNA was extracted from patients’ tumors and matched peripheral blood using the Qiagen DNA kit. Fragmented genomic DNA from tumor and normal samples was used for Illumina TruSeq library construction, and exonic regions were captured in solution using the Agilent SureSelect V4 kit according to the manufacturer’s instructions, as previously described^43,49,50^. Paired-end sequencing, resulting in 100 bases from each end of the fragments for the exome libraries, was performed using Illumina HiSeq 2000/2500 instrumentation. The mean depth of total coverage for the pre-treatment tumors and matched normal DNA samples was 220× (166× distinct) and 105× (90× distinct,) respectively (Supplementary Table 2). On average, 94% of the bases in the target region had a minimum coverage of 10×; four tumor samples (329, 351, 629 and 923) were determined to be of low purity by mutation and copy number analyses and were excluded from all WES-based analyses of somatic alterations, whereas their matched normal DNA samples were included in the germline analyses.

### Somatic mutation calling, immunogenic mutation characterization and neoantigen prediction

Somatic mutations, consisting of point mutations, insertions and deletions, across the whole exome were identified using the VariantDx custom software for identifying mutations in matched tumor and normal samples, as previously described^43,49^. Mutations were annotated with the number of tumor samples harboring identical amino acid changes in cosmic database (v91). Somatic sequence alterations are listed in Supplementary Table 3. Sequence alterations in DNA damage repair genes were analyzed separately, and the list of DNA damage repair genes considered is shown in Supplementary Table 10. MHC class I and II neoantigens were derived from non-synonymous single-base substitutions using MHCnuggets^51^. Ranks of neopeptides were determined based on their MHC binding affinity compared to 10,000 human proteome peptides per peptide length per binding MHC allele. Sequence alterations resulting in neopeptides ranking in the 1st percentile were considered putatively IMMs (Supplementary Table 3).

### Germline predisposition characterization

A set of cancer susceptibility genes with alterations contributing to germline predisposition to mesothelioma was compiled from the literature^3^. Non-synonymous germline alterations in the above set were identified by applying Strelka 2.9.2 (ref. ^52^), and the candidate mutation set was first filtered to include positions where the genotype was of sufficient quality and could be resolved in both normal and tumor samples of each patient. Variants were subsequently annotated using OpenCRAVAT^53^. Confirmed pathogenic variants—hereafter termed germline deleterious mutations—including nonsense, frameshift, splice site and missense variants, in genes with known cancer susceptibility potential, were identified based on annotation in the ClinVar database and published evidence of a damaging effect on protein function.

### Mutation signatures

Mutation signatures were derived based on the fraction of coding point mutations in each of 96 trinucleotide contexts and estimated the contribution of each signature to each tumor sample using the deconstructSigs R package (v1.8.0) with the default ‘signatures.nature2013’ settings^54^ (https://cran.r-project.org/package=deconstructSigs).

### HLA germline and somatic analyses

OptiType v1.2. was used to determine HLA class I haplotypes^55^; xHLA was used to determine HLA class II haplotypes for HLA-DPB1, HLA-DQB1, HLA-DRB1^56^; and SOAP-HLA was used to determined class II haplotypes for HLA-DPA1 and HLA-DQA1 (ref. ^57^). A separate bioinformatic analysis using POLYSOLVER^58^ was used to detect and annotate the somatic mutations in class I HLA genes. We determined HLA class I loss in the tumor by applying LOHHLA^59^. We evaluated somatic loss of HLA class II genes by review of allele-specific copy number of these loci, where minor copy number of 0 indicated LOH. The number of unique tumor HLA class I and II alleles was calculated by subtracting the number of heterozygous alleles with somatic LOH from the total number of unique germline alleles. We subsequently computed an HLA evolutionary divergence (HED) score by using Grantham distances between protein sequences of allele pairs for each HLA-A, HLA-B and HLA-C locus^25^. HLA class I allele protein sequences are obtained from the ImMunoGeneTics /HLA database^60^. A cumulative HED score for each sample was also computed as the arithmetic mean of the three individual divergences, assuming equal contribution from each locus. Germline and somatic HLA class I and II genomic variation is summarized in Supplementary Table 5.

### Genome-wide copy number analyses

We used FACETS 0.6.1 to estimate the purity of each tumor sample, the integer allele-specific copy number profile across the genome and the cellular fraction associated with each aberrant somatic copy number alteration^61^. The estimated allele-specific copy number profiles were reviewed to ensure quality of fit. In four cases with very low tumor purity (329, 351, 629 and 923), the copy number states and ploidy could not be resolved; these cases were excluded from subsequent copy-number-based analyses. Furthermore, we investigated potential associations between copy-number-derived tumor purity and tumor mutation and IMM load; these analyses revealed a weak association between tumor purity and TMB-derived features when all patients were considered, but no statistically significant association was observed among these features in epithelioid mesotheliomas (Extended Data Fig. 10). Three cases (225, 926 and 922) harbored extensive LOH across the genome with evidence of GNH (Fig. 4 and Extended Data Fig. 9). In each tumor sample, the number of sequence alterations overlapping loci with total copy number of 1 was recorded. Focal copy number changes—that is, amplifications and deletions—were determined as genomic regions of a size smaller than 30 Mb where the assigned copy number was 0 (homozygous deletion), or it exceeded three times the ploidy of the tumor sample (amplifications; Supplementary Table 7). The estimated ploidy was rounded to the closest integer level and was used as the reference for determining loss, gain or neutral status of each copy number segment. Gain, loss or LOH in each chromosome arm was evaluated if at least 90% of the length of the arm was covered by segments of the given status. The statistical significance of the prevalence of each category of alterations across the arms was evaluated by performing a permutation experiment. In this experiment, each permutation sample was a vector of size 39 where the total number of chromosome arms harboring gain, loss or LOH equaled this value for one of the samples in the main cohort to match the observed level of aneuploidy in the population.

### Mutation clonality estimation

For each somatic sequence alteration, the observed mutant and total read counts, the tumor purity and the tumor copy number at the mutated locus were integrated using SCHISM^62^ and as previously described^50^ to determine the clonality—that is, the fraction of cancer cells that harbor the alteration (Supplementary Table 3).

### Aneuploidy assessment

Several metrics characterizing the degree of genome aneuploidy were calculated, including the fraction of genome with LOH, the fraction of genome with allelic imbalance, the number of copy number breakpoints and the entropy of the multinomial probability distribution corresponding to the genome representation of different copy number levels (Supplementary Table 7)^50^. The number of copy number breakpoints was used as a proxy measure for the extent of somatic structural alterations in each tumor.

### Homologous recombination deficiency estimation

To assess the extent of HRD in tumors, three individual and one combined metric were determined based on the allele-specific copy number profiles by applying the R package scar-HRD 0.1.1 (ref. ^63^): telomeric allelic imbalance (HRD-TAI score; the count of copy number segments with allelic imbalance that extend of telomeres); loss of heterozygosity profiles (HRD-LOH score; the number of segments with a minimum size of 15 Mb that do not span the entire chromosome); and large-scale state transitions (HRD-LST score; the number of breakpoints between segments with minimum size of 10 Mb where the gap between the segments does not exceed 3 Mb). A combined metric for HRD, termed HRD-sum, was defined as the sum of the three individual metrics (Supplementary Table 7).

### Evaluation of the background rate of genomic loss

To better characterize the background rate of loss in regions of the genome with a single copy per cell (haploid) versus euploid regions (two copies per cell, no LOH), we analyzed somatic copy number profiles of 1,086 mesothelioma and non-small-cell lung cancer tumors from TCGA. In each tumor, we first determined the chromosome arms where at least 75% of the arm length was covered by the copy number state of interest. The set of tumor samples was then narrowed down to those with at least one arm in haploid state (*n* = 544). Next, across all chromosome arms of a given state, the rate of loss was determined as follows. In the haploid arms, the loss rate was defined as the total number of bases with somatic copy number of 0 within these arms, divided by the total length of arms in haploid state. In the diploid arms, the loss rate was defined as the total number of bases with somatic copy number of 0 within these arms multiplied by 2 added to the number of bases with somatic copy number of 1, and then divided by the total length of arms in euploid state.

### TCR sequencing

Intra-tumoral TCR clones were evaluated by next-generation sequencing of the baseline tumor as well as matched baseline-resistant tumors for cases 295, 459 and 926. TCR-β CDR3 regions were amplified using the survey ImmunoSeq assay in a multiplex PCR method using 45 forward primers specific to TCR Vβ gene segments and 13 reverse primers specific to TCR Jβ gene segments (Adaptive Biotechnologies)^64^. Productive TCR sequences were further analyzed, and clone counts were based on CDR3 amino acid sequences (Supplementary Table 9). Dominant TCR clones were assessed by estimating the proportion of TCR repertoire constituted by the top 5% of unique clones; for these analyses, TCR repertoires were filtered for clones representing at least 0.01% of the repertoire. For each sample, a clonality metric was estimated to quantitate the extent of mono- or oligo-clonal expansion by measuring the shape of the clone frequency distribution. For differential abundance analysis between baseline and on-therapy tumors, we selected the most expanded and most regressed TCR clonotypes, corresponding to fold changes in productive frequency of TCR clones with a false discovery rate (FDR) < 0.01 (Fisher’s exact test) and requiring at least 0.01% relative repertoire abundance at baseline or resistance time points.

### RNA sequencing

Total RNA was extracted from 10-µm FFPE sections with the RNeasy FFPE kit (Qiagen). The quality of total RNA was assessed by calculating the DV200 index measured with the RNA 6000 Pico Kit (Agilent Technologies). RNA sequencing (RNA-seq) libraries were generated by ribosomal depletion (Illumina Ribo-Zero Gold rRNA Removal Kit) followed by reverse transcription into strand-specific cDNA libraries (NEBNext Ultra Drectional RNA Library Prep kit for Illumina). Paired-end sequencing, resulting in 150 bases from each end of the fragments, was performed using Illumina NovaSeq 6000 S4, generatig an average of 200 million total reads per library. RNA-seq data were then mapped to the human transcriptome using STAR^65^ followed by RSEM for isoform and gene-level quantification^66^. Transcripts associated with RNA genes and mitochondrial genes and with ribosomal proteins were masked. Normalization of raw transcript counts and differential expression analysis was performed using DESeq2 (ref. ^67^).

### PD-L1 and CD8 immunohistochemistry

Immunohistochemistry for CD8/PD-L1 dual detection was performed on FFPE sections on a Ventana Discovery Ultra autostainer (Roche Diagnostics) using a primary mouse anti-human CD8 antibody (1:100 dilution, clone m7103, Dako) and a rabbit anti-human anti-PD-L1 antibody (1:100 dilution, E1L3N clone, Cell Signaling Technologies), as previously described. A minimum of 100 tumor cells were evaluated per specimen, and a PD-L1 tumor proportion score was calculated based on the percentage of tumor cells with PD-L1-positive staining. CD8-positive lymphocyte density was evaluated by the average number of CD8^+^ cells in ten representative high-power fields (×40 objective, ×400 magnification).

### Functional T cell assays

To identify immunogenic, mutation-derived, neopeptide-specific, HLA class I-restricted T cell clones in the peripheral blood, we applied the high-throughput TCR-seq-based platform MANAFEST (Mutation-Associated NeoAntigen Functional Expansion of Specific T Cells), as previously described^43,68^. In brief, putative neopeptides identified above (JPT Peptide Technologies; Supplementary Table 11) were each used to stimulate 250,000 T cells in vitro for 10 d. On day 0, T cells were isolated from peripheral blood mononuclear cells by negative selection (EasySep, STEMCELL Technologies). The T-cell-negative fraction was co-cultured with an equal number of selected T cells in culture medium (IMDM/5% human serum with 50 μg ml^−1^ of gentamicin) with 1 μg ml^−1^ of relevant neoantigenic peptide, 1 μg ml^−1^ of an MHC class I-restricted CMV, EBV and flu peptide epitope pool (CEFX, JPT Peptide Technologies), 1 μg ml^−1^ of pools representing the HIV-1 Gag protein (JPT Peptide Technologies) and no peptide. On day 3, half of the medium was replaced with fresh medium containing cytokines for a final concentration of 50 IU ml^−1^ of IL-2 (Chiron), 25 ng ml^−1^ of IL-7 (Miltenyi) and 25 ng ml^−1^ of IL-15 (PeproTech). On day 7, half of the medium was replaced with fresh culture medium containing cytokines for a final concentration of 100 IU ml^−1^ of IL-2 and 25 ng ml^−1^ of IL-7 and IL-15. On day 10, cells were harvested and washed twice with PBS, and the CD8^+^ fraction was isolated using a CD8^+^-negative enrichment kit (EasySep, STEMCELL Technologies). DNA was extracted from each CD8-enriched culture condition. TCR Vβ CDR3 sequencing was performed by the SKCCC FEST and TCR Immunogenomics Core (FTIC) on genomic DNA from each T cell condition using the Oncomine TCR Beta short-read assay (Illumina). DNA libraries were pooled and sequenced on an Illumina iSeq 100 using unique dual indexes to prevent index hopping, with an estimated recovery of ~50,000 reads per sample. Data pre-processing was performed to eliminate non-productive TCR sequences (sequences that did not translate into a productive protein) and to align and trim the nucleotide sequences to obtain only the CDR3 region. Additionally, for inclusion in our analyses, CDR3 sequences needed to begin with ‘C’, end with ‘F’ or ‘W’ and have at least seven amino acids in the CDR3, which are universally accepted parameters for delineating the CDR3 region^69^. Productive clonality of each sample and productive frequency of each clone was calculated to reflect the processed data (Supplementary Table 12). Resultant processed data files were uploaded to our publicly available MANAFEST analysis web app (www.stat-apps.onc.jhmi.edu) to bioinformatically identify neoantigen-specific T cell clonotypes. To be considered antigen-specific, a T cell clonotype must have met the following criteria: (1) significant expansion (Fisher’s exact test with Benjamini–Hochberg correction for FDR, *P* < 0.05) compared to T cells cultured without peptide; (2) significant expansion compared to every other peptide-stimulated culture (FDR < 0.05); (3) an odds ratio greater than 5 compared to all other conditions; (4) at least 30 reads in the ‘positive’ well; and (5) at least 2× higher frequency than background clonotypic expansions as detected in the HIV-negative control condition (Supplementary Tables 13 and 14).

### Statistical analyses

OS and PFS distributions were estimated using the Kaplan–Meier method, and Cox proportional hazard models were used to estimate the HRs among subgroups. The CIs of ORR (defined as the percentage of patients achieving CR or PR) were calculated based on an exact binomial distribution. ORRs were compared among subgroups using Fisher’s exact test. Differences in genomic and molecular features between tumors of responding and non-responding patients were evaluated using the chi-squared test or Fisher’s exact test for categorical variables and the Mann–Whitney test for continuous variables. The Pearson correlation coefficient (*R*) was used to assess correlations between continuous variables, and the Spearman ρ coefficient was calculated for non-parametric correlations. We investigated potential correlations between the genomic features described, and other than the expected co-linearity between non-synonymous mutation burden and MHC class I and II mutation-associated neoantigens, we did not identify any potential confounding relationships among features (Extended Data Fig. 10). The median point estimates and 95% CIs for PFS and OS were estimated by the Kaplan–Meier method, and survival curves were compared by using the non-parametric log-rank test. For the survival analyses of the TCGA mesothelioma cohort, progression-free interval was defined as the time interval from diagnosis to progression of disease, local recurrence, distant metastasis or death, whichever was applicable. Statistical analyses were performed using SPSS software (version 25.0.0 for Windows, IBM), SAS (version 9.4) and R version 3.2 and higher (http://cran.r-project.org).

### Reporting Summary

Further information on research design is available in the Nature Research Reporting Summary linked to this article.

## Online content

Any methods, additional references, Nature Research reporting summaries, source data, extended data, supplementary information, acknowledgements, peer review information; details of author contributions and competing interests; and statements of data and code availability are available at 10.1038/s41591-021-01541-0.

## Supplementary information

Reporting Summary

Supplementary Tables Supplementary Table 1. Summary of clinical features of samples analyzed. Supplementary Table 2. Summary of WES characteristics. Supplementary Table 3. Summary of somatic sequence alterations. Supplementary Table 4. Differential enrichment analysis for genomic and TCR features for all patients and patients with epithelioid MPM. Supplementary Table 5. HLA class I and II germline and somatic diversity. Supplementary Table 6. Summary of somatic focal copy number alterations. Supplementary Table 7. Genome-wide aneuploidy characteristics. Supplementary Table 8. Mutation cellularity of mutations in haploid regions for mesotheliomas in the PrE0505 cohort. Supplementary Table 9. TCR sequencing characteristics. Supplementary Table 10. List of genes involved in DNA damage response assessed in the study. Supplementary Table 11. Neopeptides tested for patients 295 and 459. Supplementary Table 12. TCR sequencing characteristics for the MANAFEST assays. Supplementary Table 13. Frequency of neopeptide-specific TCR clonotypes in each experimental condition for patient 295. Supplementary Table 14. Frequency of neopeptide-specific TCR clonotypes in each experimental condition for patient 459.

## Data Availability

All requests for raw and analyzed data and materials are promptly reviewed by PrECOG and Johns Hopkins University to verify if the request is subject to any intellectual property or confidentiality obligations. Patient-related data not included in the paper were generated as part of clinical trials and might be subject to patient confidentiality. All raw sequencing data, used to generate Figs. 2–5 and Extended Data Figs. 4–10, have been deposited in the European Genome-phenome Archive (accession number EGAS00001005426). Source data for Figs. 1–5 and Extended Data Figs. 1–10 are provided with the paper, in the Supplementary Tables and in Source Data files. Source data for the TCGA tumor samples were retrieved from http://cancergenome.nih.gov. WES-derived somatic mutation calls from the TCGA PanCancer Atlas MC3 project were retrieved from the NCI Genomic Data Commons (https://gdc.cancer.gov/about-data/publications/mc3-2017). Source data are provided with this paper.

## Source data

Source Data Fig. 1 Statistical Source Data
Source Data Fig. 2 Statistical Source Data
Source Data Fig. 4 Statistical Source Data.
Source Data Fig. 5 Statistical Source Data.
Source Data Extended Data Fig. 3 Statistical Source Data
Source Data Extended Data Fig. 8 Statistical Source Data
Source Data Extended Data Fig. 9 Statistical Source Data
