# Supplementary material for: Durvalumab with platinum-pemetrexed for unresectable pleural mesothelioma: survival, genomic and immunologic analyses from the phase 2 PrE0505 trial
Source: Nat Med. 2021 Nov 8;27(11):1910–20. doi: 10.1038/s41591-021-01541-0 (PMC8604731; doi:10.1038/s41591-021-01541-0)
Supplement: Supplementary file 1 — Reporting Summary [file 41591_2021_1541_MOESM1_ESM.pdf]

nature research

Corresponding author(s): Forde P, Anagnostou V

Last updated by author(s): Sep 15, 2021

Reporting Summary

Nature Research wishes to improve the reproducibility of the work that we publish. This form provides structure for consistency and transparency in reporting. For further information on Nature Research policies, see our Editorial Policies and the Editorial Policy Checklist.

Statistics

For all statistical analyses, confirm that the following items are present in the figure legend, table legend, main text, or Methods section.

n/a	Confirmed	
☐	☒	The exact sample size (*n*) for each experimental group/condition, given as a discrete number and unit of measurement
☐	☒	A statement on whether measurements were taken from distinct samples or whether the same sample was measured repeatedly
☐	☒	The statistical test(s) used AND whether they are one- or two-sided<br>*Only common tests should be described solely by name; describe more complex techniques in the Methods section.*
☐	☒	A description of all covariates tested
☐	☒	A description of any assumptions or corrections, such as tests of normality and adjustment for multiple comparisons
☐	☒	A full description of the statistical parameters including central tendency (e.g. means) or other basic estimates (e.g. regression coefficient) AND variation (e.g. standard deviation) or associated estimates of uncertainty (e.g. confidence intervals)
☐	☒	For null hypothesis testing, the test statistic (e.g. *F*, *t*, *r*) with confidence intervals, effect sizes, degrees of freedom and *P* value noted<br>*Give P values as exact values whenever suitable.*
☒	☐	For Bayesian analysis, information on the choice of priors and Markov chain Monte Carlo settings
☒	☐	For hierarchical and complex designs, identification of the appropriate level for tests and full reporting of outcomes
☐	☒	Estimates of effect sizes (e.g. Cohen's *d*, Pearson's *r*), indicating how they were calculated

Our web collection on statistics for biologists contains articles on many of the points above.

Software and code

Policy information about availability of computer code

Data collection	Clinical data were collected utilizing the RAVE electronic data capture (eDC) system QDR3.
Data analysis	Statistical analyses were done using SAS (version 9.4), SPSS software program (version 25.0.0 for Windows, IBM) and R version 3.2 and higher, http://www.R-project.org/).

For manuscripts utilizing custom algorithms or software that are central to the research but not yet described in published literature, software must be made available to editors and reviewers. We strongly encourage code deposition in a community repository (e.g. GitHub). See the Nature Research guidelines for submitting code & software for further information.

Data

Policy information about availability of data

All manuscripts must include a data availability statement. This statement should provide the following information, where applicable:

- Accession codes, unique identifiers, or web links for publicly available datasets
- A list of figures that have associated raw data
- A description of any restrictions on data availability

All requests for raw and analyzed data and materials are promptly reviewed by PrECOG, LLC and the Johns Hopkins University to verify if the request is subject to any intellectual property or confidentiality obligations. Patient-related data not included in the paper were generated as part of clinical trials and may be subject to patient confidentiality. All raw sequencing data, utilized to generate Figures 2-5 and Extended Data Figures 4-10 have been deposited in the European Genome-phenome Archive (EGA accession number EGAS00001005426). Source data for Figures 1-5 and Extended Data Figures 1-10 are provided with the paper, in the Supplementary Tables and in Source Data files. Source data for the TCGA tumor samples were retrieved from http://cancergenome.nih.gov. WES-derived somatic mutation calls from the TCGA PanCancer Atlas MC3 project were retrieved from the NCI Genomic Data Commons (https://gdc.cancer.gov/about-data/publications/mc3-2017).

Field-specific reporting

Please select the one below that is the best fit for your research. If you are not sure, read the appropriate sections before making your selection.

☒ Life sciences ☐ Behavioural & social sciences ☐ Ecological, evolutionary & environmental sciences

For a reference copy of the document with all sections, see nature.com/documents/nr-reporting-summary-flat.pdf

Life sciences study design

All studies must disclose on these points even when the disclosure is negative.

Sample size	Using a one-sided 0.10 level test, with 50 patients, we will have 90% power to detect a 37% reduction in the OS hazard rate of 0.058 to 0.037 (with an accrual period of 24 months and an additional 18 months for treatment and follow-up); assuming exponential survival, this corresponds to a 58% improvement in the median OS of 12 months to 19 months. After inflating for an ineligibility rate of 10%, the total planned accrual is 55 patients.
Data exclusions	No data was excluded from the analysis.
Replication	This is a clinical trial. No replication was done.
Randomization	This is a single arm phase II study,no randomization was done.
Blinding	This is a single arm phase II study, no blinding was done.

Reporting for specific materials, systems and methods

We require information from authors about some types of materials, experimental systems and methods used in many studies. Here, indicate whether each material, system or method listed is relevant to your study. If you are not sure if a list item applies to your research, read the appropriate section before selecting a response.

Materials & experimental systems

n/a	Involved in the study	
☐	☒	Antibodies
☒	☐	Eukaryotic cell lines
☒	☐	Palaeontology and archaeology
☒	☐	Animals and other organisms
☐	☒	Human research participants
☐	☒	Clinical data
☒	☐	Dual use research of concern

Methods

n/a	Involved in the study	
☒	☐	ChIP-seq
☒	☐	Flow cytometry
☒	☐	MRI-based neuroimaging

Antibodies

Antibodies used	Primary mouse anti-human CD8 antibody, (Dako, catalog number m7103, clone C8/144B, 1:100 dilution) and anti-PD-L1 primary antibody (Cell Signaling Technologies, catalog number 13684, E1L3N clone, 1:100 dilution).
Validation	SDS-PAGE analysis of immunoprecipitates formed between lysates of 125I-labeled human T lymphoblasts and the antibody shows reaction primarily with a 32 kDa polypeptide corresponding to CD8a (Mason et al., J Clin Pathol 1992:45:1084-8). For the PDL1 antibody Western blot analysis of extracts from KARPAS-299, SUP-M2, and PC-3 cells using PD-L1 (E1L3N) Rabbit mAb detects the PDL1 protein as per the manufacturer's product insert.

Human research participants

Policy information about studies involving human research participants

Population characteristics	Eligible patients must have histologically and/or cytologically confirmed unresectable malignant pleural mesothelioma with age >=18 and have ECOG Performance Status of 0 or 1. Patients of either sex were included and all demographic characteristics are described in detail in Table 1 of this manuscript.
Recruitment	Patients met all of the eligibility requirements described in the protocol prior to registration. Upon determination that a patient met eligibility criteria, the patient was registered in the study by site personnel via an electronic data capture (eDC) system. To capture a representative population of patients with mesothelioma, patients were recruited at 15 centers across the United States. These included both community and academic centers which with the goal of reducing selection bias, the eligibility criteria (noted in the manuscript) were also developed to minimize bias.

Ethics oversight	The United States Food and Drug Administration; HealthPartners Institute Park Nicollet Health System Institutional Review Board.

Note that full information on the approval of the study protocol must also be provided in the manuscript.

Clinical data

Policy information about clinical studies

All manuscripts should comply with the ICMJE guidelines for publication of clinical research and a completed CONSORT checklist must be included with all submissions.

Clinical trial registration	NCT02899195
Study protocol	The clinical trial protocol is included with this submission.
Data collection	Data were collected at the 15 participating cancer centers (academic and community) in the United States. The trial was activated on May 1, 2017 and completed accrual on June 21, 2018.
Outcomes	The primary endpoint of the study was overall survival. Overall survival was defined as the time from randomization to death from any cause. Patients that did not have an event reported at the time of analysis were censored at their date of last follow-up. Secondary endpoints included progression-free survival (PFS), best objective response, and toxicity. PFS was defined as the time from randomization to documented disease progression or death from any cause, whichever occured first. Patients who did not experienced an event of interest by the time of analysis were censored at the date they are last known to be alive and progression-free. Best objective response was evaluated via RECIST Version 1.1 criteria modified for mesothelioma. Toxicity was determined using the CTCAE Version 4.03 criteria.
